# Advayavajra’s *Nairātmyāprakāśa*: A Critical Edition

**DOI:** 10.1007/s10781-025-09611-0

**Published:** 2025-06-16

**Authors:** Ryan Conlon

**Affiliations:** Universitāt Hamburg, Hamburg, Germany

**Keywords:** Advayavajra, Maitrīpāda, Nairātmyā, Hevajratantra, Tantra, Sādhana, Vajrayāna Buddhism

## Abstract

This article presents a critical edition of and commentary on the *Nairātmyāprakāśa*, a Buddhist tantric ritual manual (*sādhana*) composed by the eleventh-century scholar and *siddha* Advayavajra/Maitrīpāda. The text teaches meditations on Nairātmyā, the central *yoginī* of the Hevajra system, while also incorporating distinctive elements of Advayavajra’s philosophy and system of tantric practice. An introduction to the edition examines the figure of Nairātmyā and her associated corpus of texts. It also situates the *Nairātmyāprakāśa* within Advayavajra’s broader oeuvre and identifies traces in this text of the author’s intellectual and religious ecosystem, particularly in light of his textual borrowings from Ratnākaraśānti.

## Introduction

This^1^ article presents an annotated critical edition of the *Nairātmyāprakāśa*, a *sādhana* by the famed Indian scholar and *siddha* Advayavajra (early- to mid-eleventh century),^2^ also known as Maitrīpāda, Maitreyanātha, and Maitrīgupta. This ritual manual is one of the few extant Sanskrit texts that outline a meditation on the goddess Nairātmyā, the principal *yoginī* in the system of the *Hevajratantra*. Advayavajra’s *sādhana* is significant for its clear elucidation of the author’s approach to deity yoga, as well as for its inclusion of his broader schematization of Buddhist tantric practice. The *sādhana* also betrays some of Advayavajra’s major influences, particularly through its incorporation of phraseology borrowed directly from Ratnākaraśānti, said to have been one of his teachers.

By way of introduction, I provide a brief overview of Nairātmyā and her *sādhana*s, as well as of Advayavajra’s ritual texts. I examine distinctive features of the *Nairātmyāprakāśa* in relation to other *sādhana*s attributed to him, while also highlighting traces of his intellectual and religious ecosystem. The critical edition follows, accompanied by an apparatus and detailed notes that clarify editorial decisions and textual interpretation.

Nairātmyā is the central *yoginī* of the *Hevajratantra*, consort of the eight-faced (*aṣṭāsya*) Heruka taught therein and also the principal figure (*nāyikā*) of her own *maṇḍala*. Her most frequently used appellation in the *Hevajratantra* is Nairātmyayoginī, which is well suited for the end of an even *pāda* of *anuṣṭubh* verse, and of which the name Nairātmyā can easily be seen as a contraction (just as Satyabhāmā is contracted to Satyā).^3^ She is also referred to by the names Nirātmikā and Nairātmikā, as well as with the orthographic variant Nairātmā commonly found in manuscripts. These names express the Buddhist doctrine of ‘no self’ and thus the related doctrines of ‘insight’ (*prajñā*) and ‘emptiness’ (*śūnyatā*), all of which, in tantric Buddhism, are closely associated with feminine imagery.

Nairātmyā appears as a black or dark blue (*kṛṣṇa*) figure with two arms, one head, and three eyes, adorned with ornaments of skulls and bones. She holds a chopper (*kartrī*, or, commonly in scripture, *karti*) and a skull in her hands, with the chopper also serving as her insignia (*cihna*) which manifests from her seed syllable *a*. Of the Buddhist *yoginī*s, it would appear that Vajrayoginī and Vajravārāhī of the *Cakrasaṃvara* system enjoyed greater popularity in both India and Tibet, evidenced in part by the number of extant ritual manuals associated with these goddesses. Nonetheless, the two systems were by no means sealed off from each other: the *Hevajratantra* itself, for instance, names Vajravārāhī as the consort of the four-armed Heruka, and later texts of the *Cakrasaṃvara* system, such as the *Ḍākārṇavatantra*, also refer to the goddess Nairātmyā.^4^

The principal scriptural sources for Nairātmyā are the *Hevajratantra* (especially the eighth chapter of the first section, which teaches her *maṇḍala*), the *Vajrapañjaratantra*, and the *Saṃpuṭatantra*. A handful of Indic ritual texts dedicated to Nairātmyā, including both *sādhana*s and *stuti*s, are either still extant in their original language or available in Tibetan translation. To my knowledge, the former are mostly transmitted in two *sādhana* collections: nos. 228–231 in the *Sādhanamālā* as edited by Bhattacharyya, and nos. 37–44 in the collection sometimes called the *Hevajrasādhanasaṅgraha* as described by Isaacson (2009). There are also at least seven presumably Indic texts now preserved only in Tibetan translations.^5^

**Table:** 

Sanskrit source	Title and author (if applicable)	Tibetan bsTan ’gyur
SāMā 228, HeSāSa 39	*Amṛtaprabhā Sādhanopāyikā* attributed to Ḍombīheruka	Tōh. 1305
SāMā 229	*Nairātmyāmaṇḍalayoginīviśuddhi*	
SāMā 330	*Kevalanairātmyāsādhana*	Tōh. 3992 and 3639
SāMā 231, HeSāSa 42	*Nairātmyāsādhana* by Sahajavilāsa	Tōh. 3294, 3393 and 3640
HeSāSa 37	*Nairātmyāśīrūpā stutiḥ*	
HeSāSa 38	*Nairātmyāstuti*	
HeSāSa 40	*Nairātmyāsādhana*	
HeSāSa 41 and in *Muktāvalī* ch. 1.8	*Nairātmyāsādhana* by Ratnākaraśānti	Tōh. 1309 and within Tōh. 1189
HeSāSa 43	*Tattvāvaloka Nairātmyāsādhana* by Divākaracandra	
HeSāSa 44	*Nairātmyāprakāśa* by Advayavajra	Tōh. 1308
	*Nairātmyāsādhana by Durjayacandra	Tōh. 1306a
	*Ekavīrāsādhana by Rāhula	Tōh. 1310
	*Nairātmyāsādhana by Kṛṣṇa	Tōh. 1311
	*Nairātmikopadeśa by Samādhivajra	Tōh. 1312
	*Nairātmyāmaṇḍalacakrasādhana	Tōh. 1313
	*Pañcadaśadevīstuti by Durjayacandra	Tōh. 1307
	*Nairātmyābalacakrasādhana by *Mahāsukha	Ōta. 4708

SāMā = *Sādhanamālā*; HeSāSa = *Hevajrasādhanasaṅgraha*

Tibetan authors also composed *sādhana*s of Nairātmyā, though these too are not overly numerous. A notable example is Chos rgyal ’Phags pa Blo gros rgyal mtshan’s (1235–1280) *bDag med lha mo bco lnga’i mngon rtogs*, written at the request of the Mongolian prince Zhenjin in 1273. Another exemplary text is Tsong kha pa Blo bzang grags pa’s (1357–1419) *bDag med ma’i dkyil ’khor gyi sgrub thabs zin bris*, a work that treats a variety of the Indic sources by referencing Saroruhavajra, Ḍombīheruka, Durjayacandra, Ratnākaraśānti, and Abhayākaragupta. It also engages with the Sa skya pas and the followers of Mar pa Chos kyi blo gros. Zhwa dmar bzhi pa Chos grags ye shes’s (1453–1524) *bDag med ma’i sgrub thabs rmongs pa’i mun sel* is significant for its influence on the now widely used manuals of Kong sprul Blo gros mtha’ yas (1813–1899). The latter composed a short and long *sādhana* as well as an initiation manual in his *bKa’ brgyud sngags mdzod*.

Advayavajra is one of the key figures of late Indian tantric Buddhism as a teacher, an author, and, in subsequent generations, a lineage patriarch for certain Tibetan Buddhists.^6^ It is clear that he composed many texts, and a series of them came to be known in Tibet as the *Cycle of Teachings on Amanasikāra* (*yid la mi byed pa’i chos skor*), mostly corresponding to the collection of texts first edited by Bhattacharyya in 1927 and known as the *Advayavajrasaṅgraha*. These texts are short and pithy; at times, owing to either their poor transmission or their being originally intended for members of the author’s inner circle, they can also be described as obscure. Apart from this collection, a large number of texts, all essentially tantric in orientation, are associated with the name of Advayavajra or another of his aliases. Isaacson and Sferra (2014, pp. 72–75) have discussed such works that are still extant in Sanskrit, but a systematic study of those only available in Tibetan translation, which by my count number at least eighty-one,^7^ remains a scholarly desideratum.

Isaacson and Sferra (2014, pp. 73–74) identify the following seven *sādhana*s available in Sanskrit as attributed to Advayavajra:

**Table:** 

Sanskrit source	Title	Tibetan translation(s)
SāMā 17	*Siṃhanādasādhana*	Tōh. 3414
SāMā 217	*Vajravārāhīsādhana*	Tōh. 3607 and Tōh. 1542
SāMā 251	*Saptākṣarasādhana*	Tōh. 1483
GuSaSāMā 15	*Sarvārthasiddhisādhana*	Tōh. 1578 and Tōh. 1552
HeSāSa 2	*Hevajrākhya*	n/a
HeSāSa 7	*Hevajraviśuddhinidhi*	Tōh. 1243 and Tōh. 1244
HeSāSa 44	*Nairātmyāprakāśa*	Tōh. 1308

SāMā = *Sādhanamālā*; HeSāSa = *Hevajrasādhanasaṅgraha*; GuSaSāMā = *Guhyasamayasādhanamālā*

Isaacson and Sferra (2014, p. 74) regard all of these works as potentially authored by Advayavajra, while highlighting some compelling internal evidence for the authorship of the *Nairātmyāprakāśa* and *Hevajraviśuddhinidhi*: namely, the former describes Advayavajra’s schematization of tantric practice, which is also mentioned in his other works; and the latter states that the *sādhana* was composed for one Ratnadevī, a figure described in Tibetan sources as the consort of Advayavajra’s student Rāmapāla (Isaacson & Sferra, 2014, 295 n. 233). As I will highlight below, there are also significant content overlaps between the two works, particularly in their phraseology and in their sometimes unique descriptions of the visualisations’ *viśuddhi*s or ‘pure natures’. Let us accept Isaacson and Sferra’s overall assessment of these seven *sādhana*s as a starting point and turn to examining features of the *Nairātmyāprakāśa*. In so doing we can gain further clarity regarding Advayavajra’s authorship of the other *sādhana*s, as well as of two *sādhana*s in Tibetan translation that stand out: the *Cakrasaṃvarasādhana (Tōh. 1484) and the closely related *Cakrasaṃvaropadeśa (Tōh. 1485).

The structure of the *Nairātmyāprakāśa* can be organised into roughly four parts: the *sādhana* preliminaries, the main process of deity visualisation, a brief description of alternative (and more advanced) Nairātmyā-oriented practices, and a list of mantras. The text also begins and ends with the kind of verses conventionally found in such Sanskrit compositions. Its opening benediction can be described as a signature verse of Advayavajra, praising Vajrasattva with reference to his three *kāya*s. The verse is also found in the two Cakrasaṃvara *sādhana*s just mentioned, as well as in the Tibetan translation of the *Saptākṣarasādhana*. Following a description of the *sādhaka*’s required qualifications, the *Nairātmyāprakāśa* continues with the standard preliminaries of taking refuge, confessing sins, beseeching the enlightened ones, and so forth. The phraseology of this passage, while formulaic in character, has particularly close parallels with the same procedures in the *Vajravārāhīsādhana* and the *Saptākṣarasādhana*.

At the start of the main visualisation sequence, when the practitioner is to engage with ultimate reality, Advayavajra introduces an allusion to another of his signatures—namely, the form of Madhyamaka philosophy that he termed *sarvadharmāpratiṣṭhānavāda* (‘the doctrine of the non-abiding of all phenomena’). The *sādhaka* should ‘rest in a way such that he does not remain fixed anywhere’ (*apratiṣṭhitarūpeṇa tiṣṭhet*) as he focuses on a mantra that expresses the identity of the practitioner with non-dual knowledge and the emptiness of all phenomena. Almost identical descriptions of this contemplation and mantra are put forward in the *Vajravārāhīsādhana*, the *Saptākṣarasādhana*, the *Hevajraviśuddhinidhi*, and the two Cakrasaṃvara *sādhana*s (detailed references are given below, in the notes to the edition). While its omission from his shorter works, such as the *Sarvārthasiddhisādhana*, is understandable, its absence from the otherwise very detailed *Hevajrākhya* is a factor to consider when assessing that text’s authorship.^8^ This emphasis on the emptiness of all phenomena and *apratiṣṭhāna* contrasts strikingly with *sādhana* authors who offer more generic descriptions of ultimate reality at this point in their texts, and even more so with Ratnākaraśānti’s manuals such as the *Bhramaharasādhana* (pp. 158–159) and *Mahāmāyāsādhana* (pp. 133–144), which emphasise that author’s own unique formulation of Yogācāra philosophy.

The majority of the *Nairātmyāsādhana* consists of the kind of practice Advayavajra and most tantric Buddhists call the *utpattikrama*^9^ or ‘stage of arising’, which involves the practitioner visualising the manifestation of *maṇḍala* deities while identifying as their central figure, empowering them with their ultimate forms, the *jñānasattva*s, and correlating these manifest forms with aspects of awakening *qua* their *viśuddhi*s or pure natures. Near the conclusion of the text, Advayavajra delineates four alternative practices that can be applied to Nairātmyā. These are, in sequence, the *gambhīrotpattikrama* (‘profound stage of arising’), *utpannakrama* (‘stage of the arisen’), *pariniṣpannakrama* (‘stage of the complete’), and *krama* that is *svābhāvika* or ‘the inherent stage’.

Individually these five terms likely predate Advayavajra. The words *utpatti*-, *utpanna*-, and *pariniṣpannakrama* are widely attested from the early ninth century onwards, while the terms *gambhīrotpattikrama* and *svābhāvikaḥ kramaḥ* are more rare. Notably, the latter two appear to be used in a work attributed to Saraha, the so-called *Vākkośarucirasvaravajragīti or *gSung gi mdzod ’jam dbyangs rdo rje’i glu* (fol. 114v and 113v, respectively).^10^ However, the use of these five terms together to denote a schematisation of tantric practice likely originates with Advayavajra, who also articulates this system in his *Caturmudropadeśa (p. 387 ff.). The system is subsequently treated in Divākaracandra’s *Prajñājñānaprakāśa (fol. 91r), Rāmapāla’s *Sekanirdeśapañjikā* (p. 201), *Kāropāda’s *Caturmudrānvayaṭīkā (fol. 284r), and Vajrapāṇi’s *Guruparamparakramopadeśa (fol. 170r etc.). All four authors are said to be direct students of Advayavajra.^11^ The specific characteristics of the system, the reasons for its introduction, and its broader implications are rather complex matters and can hopefully be further explored in the future.

Advayavajra’s hagiographies describe him as having studied with the Buddhist luminaries of the early eleventh century: Nāropāda, Ratnākaraśānti, and Jñānaśrīmitra (Isaacson & Sferra, 2014, 63 ff.). His main tantric master, however, was the *siddha* Śabareśvara, to whom Advayavajra composed a verse of praise at the conclusion of the *Nairātmyāprakāśa*. It is likely that many elements of the *sādhana* here—including the vocabulary of Advayavajra’s *apratiṣṭhāna* philosophy, his brief but important description of the *svābhāvikakrama*, and certain unique details of the visualisation and *viśuddhi*s—are either his own personal innovations or originate from the somewhat mythical figure of Śabareśvara. Other elements, however, as identified in detail below, clearly derive from the *Sādhanopāyikā* of Saroruhavajra, who was one of the early propagators of the Hevajra system and whose manual proved highly influential (Gerloff, 2020). The Advayavajra-attributed *Hevajrākhya* also draws extensively on this source.

The *Nairātmyāprakāśa* also contains evidence of direct borrowings from Ratnākaraśānti—specifically from the latter’s *Muktāvalī Hevajrapañjikā* in an extended passage on the *nāḍī*s and their functions. Advayavajra’s *Hevajraviśuddhinidhi* likewise borrows directly from Ratnākaraśānti’s *Bhramaharasādhana*.^12^ Although these borrowings do not prove direct personal contact between the two figures, much less a student-teacher relationship, they do reveal that while Advayavajra departed from Ratnākaraśānti on points of philosophy, he adhered closely to that scholar’s interpretation of the *Hevajratantra*, concerning both the sequence of blisses (*ānanda*s) and the details of visualisation practice.

## Introduction to the Edition

The *Nairātmyāprakāśa* is transmitted in a single Sanskrit witness, part of a 272-folio multi-text manuscript known as the *Hevajrasādhanasaṅgraha*. Containing a variety of Hevajra-related ritual texts, this manuscript and its contents have been thoroughly described by Isaacson (2009). The *Nairātmyāprakāśa* likewise has but one Tibetan translation executed by Vajrapāṇi, said to be one of Advayavajra’s students, and mTshur Ye shes ’byung gnas.^13^ The Sanskrit witness unfortunately does not transmit the text particularly well, as a result of which I have made considerably more emendations and conjectures than I would have preferred. Many of these proposals are prompted or supported by parallels in related texts (especially the *Hevajratantra* and Advayavajra’s other *sādhana*s), as well as by the Tibetan translation. The Tibetan translation can be charitably described as ‘free’, but in a number of places, as will be noted, it can only be described as mistaken. I am therefore hesitant to conjecture readings based solely on what this translation suggests. It nonetheless provides invaluable evidence for postulating what Advayavajra may have originally written.

Around the same time that I prepared an initial draft of this edition, the journal *Dhīḥ* also published an independent edition and Hindi translation of the *Nairātmyāprakāśa* by Thinlay Ram Shashni. Shashni’s publication represents a valuable contribution to the study of tantric Buddhism, as does so much of the scholarship from the Central Institute of Higher Tibetan Studies (CIHTS). All editions, however, insofar as they are hypotheses, have by definition room for reassessment and refinement, and in this case both the reporting of the manuscript readings and the constitution of the edited text can be meaningfully improved. While Shashni proposed many necessary emendations, he has also frequently let faulty manuscript readings stand, resulting in a text that is either incoherent or of implausibly poor Sanskrit for an authority such as Advayavajra. Alongside my own edition, here I also hope that a thorough examination of the textual challenges in editing this *sādhana* may prove beneficial for those wishing to engage with this text or similar ones in Sanskrit. I have not collated here the edition published in *Dhīḥ*, given that it is based on the same manuscript and makes reference to the same Tibetan translation. I do, however, reference some of its editorial decisions that are of interest in the notes.

In this edition I silently standardise orthographies and *saṃdhi* usage, while maintaining precise transcriptions of the manuscript in the apparatus. The manuscript images available to me make it particularly difficult to discern *virāma*s, so I consistently assume their presence where needed.^14^ The Tibetan translation makes only occasional appearances in the apparatus when its readings have a clear bearing on the lemma in question. Otherwise the various characteristics of the Tibetan translation are treated in the notes. Although I have transcribed a number of witnesses of the Tibetan translation, their variant readings are rarely significant for the purpose of editing the Sanskrit text. I therefore mostly refer only to the Tibetan translation as transmitted in the sDe dge edition of the bsTan ’gyur, except where a variant is especially noteworthy.

## Sigla, Symbols, and Abbreviations

**Table:** 

Ṅ	*Nairātmyāprakāśa* by Advayavajra. No. 44 in *Hevajrasādhanasaṅgraha*, fols. 260r5–264v5.
T_D_	*bDag med ma’i rab tu gsal ba* by gNyis su med pa’i rdo rje. Translation by Vajrapāṇi and Jñānākara (mTshur Ye shes ’byung gnas). Tōh. 1308, sDe dge bsTan ’gyur, vol. 10 (rGyud ’grel, Ta), fols. 218v5-223r1.
T_P_	Ōtani 2438, Pe cing bsTan ’gyur, vol. 57 (rGyud ’grel, Za), fols. 95r3–99v5.
T_N_	sNar thang bsTan ’gyur, vol. 24 (rGyud ’grel, Za), fols. 89r4–93v6.
*Dhīḥ* edition	T. R. Shashni (ed.). 2024. “Śrīmatpaṇḍitācāryāvadhūtādvayavajrapādakṛtaḥ Nairātmyāprakāśa (Hindī anuvāda sahita).” *Dhīḥ: Journal of Rare Buddhist Texts Research* 61: 113–128.
MuĀ	*Muktāvalī Hevajrapañjikā*
NaiPra	*Nairātmyāprakāśa*
HeTa	*Hevajratantra*
^ac^	*ante correctionem*
D	sDe dge
*deest*	omitted in
*diag. conj.*	diagnostic conjecture [e.g. ‘reconstructed’ from Tibetan]
*conj.*	conjecture
*em.*	emendation [an emendation is made with a high degree of confidence, whereas a conjecture proposes a correction while acknowledging a greater possibility for alternatives]
fol./fols.	folio/folios
^pc^	*post correctionem*
^r^	recto
^v^	verso
Σ_X_	Reading shared in all witnesses but X
((kiṃcit))	Reading uncertain—either illegible or otherwise in doubt
<kiṃcit>	Reading cancelled
^†^kiṃcit^†^	Reading does not make sense to the editor and an adequate conjecture was not able to be chosen.
[kiṃcit]	Indication of a diagnostic conjecture
..	Damaged *akṣara* (one . per half *akṣara*)
...	Lacuna of an unknown number of *akṣara*s
°	Mark of abbreviation

## Edition of the Sanskrit Text

oṁ namaḥ śrīnairātmyāyai |^i^

parihṛtaparikalpaṃ dharmakāyaṃ yam āhur

nirupamasukhamātraṃ cārusambhogakāyam |

bhuvanahitavidhānād yasya nirmāṇakāyaṃ

bhavatu sa bhagavān vaḥ śreyase vajrasattvaḥ ||^ii^

ekatra vitataṃ spaṣṭaṃ sabodhaṃ^15^ laghuvistaram |^iii^

nairātmyāsādhanaṃ brūmo yathāmati yathāgamam ||

yogī khalu śmaśānādau^16^ manonukūle sthāne pañcāmṛtādisamayasevī sukhāsanopavi[Ṅ fol. 260^v^]ṣṭo^17^ niḥsaṅgo niḥśaṅkaḥ^18^ sattvārthodyatamatir^19^ nairātmyāhaṃkāram utpādya,^iv^ hṛtsūrye nīla-hūṁ-kāraṃ dhyāyāt. tatas tadīyai^20^^,v^ raśmibhis traidhātukam avabhāsamānair ākṛṣya, akaniṣṭhabhuvanavarti- nam^vi^ aṣṭayoginīparivṛtaṃ ṣoḍaśabhujam aṣṭāsyaṃ kapālamālāviracitaśekharaṃ catuścaraṇasamākrāntacaturmāraṃ nīlavarṇaṃ dakṣiṇakaranikarakalitakapālasaṃkalanilīnagaja-turaga-khara-vṛṣabha-karabha-manuja-śarabha-vṛṣadaṃśam itarapāṇikadambagatapadmabhājanavartidharaṇi-varuṇa-samīraṇa-jvalana-rajanīnātha-taraṇi-yama-dhanadaṃ kṛṣṇapradhānavadanam indukundāvadātadakṣiṇamukham^vii^ atimātralohitavāmavadanam atidhūmravikarālordhvavaktram alimaline tarasakalavadanaṃ^21^^,viii^ śatārdhamuṇḍamālālaṅkṛtaṃ nairātmyāliṅgitakandharam^22^ ambaratalavartinam^23^ agrato dhyāyāt.

tadanantaraṃ bāhyaguhyatattvapūjābhir^24^ aṣṭayoginībhiḥ pūjayet. atra ca prajñopāyayostādā[tmyāvabodhanāya picuvajrasya pūjanam. tato vandanaṃ pāpadeśanāpāpākaraṇasaṃvaraṃ puṇyānumodanāpuṇyapariṇāmanātriśaraṇagamanabodhicittotpādā]tmabhāva^25^niryātanādhyeṣaṇāś^26^^,ix^ca kṛtvā, caturbrahmavihārān bhāvayitvā, sakalavastutattvasārasaṅgrāhakātmakaṃ^27^oṁ śūnyatājñānavajrasvabhāvātmako^x^’haṃ śūnyatājñānavajrasvabhāvātmakāḥ sarvadharmāḥ^xiv^[Ṅ fol. 261^r^] iti mantrārthaṃ bhāvayann apratiṣṭhitarūpeṇa tiṣṭhet.

tataḥ praṇidhim anusmṛtya, samādher vyutthāya,^xii^repheṇa purataḥ sūryamaṇḍalaṃ dhyātvā, tatratya-hūṁ-kāreṇa viśvavajraṃ ca dhyātvā, tato viśvavajrāt sphuradbhir aṃśusaṃhater vajrair^28^^,xiii^ vajraprākāraṃ^29^ pañjarabandhanam^xiv^ adho vajramayīṃ bhūmiṃ^30^ parikhāṃ ca vicintayet.^31^ raviviśvavajrābhyāṃ ca raśmībhūya samantataḥ prasṛtābhyāṃ tat sarvaṃ dṛḍhīkuryāt.^xv^

tadanantaraṃ khadhātau dharmodayākārām antaḥsuṣirām atibahaladhavalām ūrdhvaviśālāṃ^32^^,xvi^ prajñāṃ paśyet. tatas tadantarvarti viśvavarṇāṣṭadalaṃ^33^ viśālaṃ^34^ kamalaṃ dhyāyāt. tatas tanmadhye^xvii^ rephodbhavasūryamaṇḍala- madhyavarti-hūṁ-kārapariṇataṃ viśvavajraṃ cintayet. viśvavajramadhye^35^ca mārutatejojalāvanimaṇḍalāni^36^^,xviii^ dhūmraraktaśuklaharitāni dhanustrikoṇaparimaṇḍalacaturasrākārāṇi^xix^ yaṁ-raṁ-vaṁ-laṁ-pariṇatāny upary upari paśyet. etat sarvaṃ jñānamātram^37^ ākalayaṃs tatpariṇataṃ caturasraṃ caturdvāram^xx^ aṣṭastambhopaśobhitaṃ hārārdhahārabhūṣitaṃ kūṭāgāraṃ paśyet.

tataḥ prākārābhyantare ’ṣṭa śmaśānāni cintayet. atra^xxi^ pūrve devendro harītakīvṛkṣe^38^^,xxii^ mecakavarṇo^xxiii^ dantivadanaḥ. dakṣiṇe [Ṅ fol. 261^v^] yamaś cūtavṛkṣe^39^ mahiṣānanaḥ sitavarṇaḥ. paścime ’śokatarau varuṇo raktaḥ siṃhamukhaḥ.^xxiv^ uttarato bodhiśākhini kubero haritābho manuṣyamukhaḥ.

āgneyyāṃ^40^ karañjavṛkṣe^41^ vaiśvānaraḥ śuklavarṇaś chāgānanaḥ. latājaṭyāṃ^xxv^ naravāhano^xxvi^ manuṣyamukhaḥ pāṇḍur nairṛtyām. vāyavyāṃ kakubhavṛkṣe pavano mṛgānanaḥ pītaḥ. aiśānyāṃ^42^ bhūteśo vṛṣabhānanaś citro nyagrodhapādape.^xxvii^ sarve cāmī vāmakarakalitakapālā nānāstravyagradakṣiṇapāṇayo darśitapūrvārd- hakāyāḥ.

evaṃ pūrvādyaṣṭadikṣu yathākramam ananta-padma-vāsuki-mahāpadma-takṣaka-śaṃkhapāla-karkoṭa-kulikāḥ. meghāś cāṣṭau mecaka-śukla-śiti-pāṇḍu-rakta-pīta-harita-viśvavarṇāś^xxviii^ cintanīyāḥ.^xxix^ evaṃ dikpālāś cāṣṭau [prasiddhavarṇāḥ. caityāni cāṣṭau] yakṣavarṇāni^43^ boddhavyāni.^44^^,xxx^

tato maṇḍalamadhye^xxxi^ ’ṣṭadalaṃ raktakamalaṃ vicintya, tatkamalamadhyapūrvādicaturdaleṣu^xxxii^ tathā kūṭāgāracatuḥkoṇeṣu caturdvāreṣv adha ūrdhvaṃ ca pañcadaśa śavān^xxxiii^ paśyet. tadanantaraṃ śavārūḍhān ālikālipariṇata- candrasūryamadhyagatān akārādipañcadaśasvarān dhyāyāt. tataḥ a-kārādibījapariṇāmena sarvatra sabījā kartikā. tataś candrasūryasabījakartikā[Ṅ fol. 262^r^] pariṇāmena^xxxiv^nairātmy-ādipañcadaśayoginīr^45^ dhyāyāt. tatrādarśajñānavāṃś candraḥ, samatājñānavān^xxxv^sūryaḥ, tayor madhyagataṃ bījaṃ pratyavekṣaṇā, sarveṣām aikyaṃ kṛtyānuṣṭhānam, bimbaniṣpattiḥ suviśuddhadharmadhātuḥ.^xxxvi^

atra varaṭakamadhye dhyeyā^46^a-kārasvarasambhavā^47^ dveṣātmikākṣobhyamudritā vijñānaskandhātmikā^xxxvii^ prajñopāyasvarūpā bahirupāyarūpakhaṭvāṅgāliṅgitakandharā^xxxviii^ nairātmyā. pūrvādidaleṣu ā-i-ī^48^-u-svarasambhavā mohapaiśunyarāger-ṣyāsvabhāvā^49^ vairocanaratnasambhavāmitābhāmoghasiddhimudritā rūpavedanāsaṃjñāsaṃskāraskandhātmikā^50^ vajrāgaurīvārīvajraḍākinīr^51^ dhyāyāt.

tato bāhyapuṭa aiśānyādikoṇeṣu ū^52^-ṛ-ṝ-ḷ-svaraniṣpannā^53^^,xxxix^akṣobhyavairo- canaratnasambhavāmitābhamudritāḥ^54^ pṛthivyaptejovāyusvabhāvāḥ pukkasīśabarīcaṇḍālīḍombīḥ paśyet.

tataḥ pūrvādidvāreṣu ḹ-e-ai-o-svarasambhavāḥ pukkasyādimudrāmudritā^55^^,xl^ rūpaśabdagandharasasvarūpā^56^ gaurīcaurīvettālīghasmaryo bhāvyāḥ. tadanantaram^57^ adha ūrdhvaṃ ca moharāgamudrite^58^au-aṃ-svarasambhave sparśadharmadhātusvabhāve bhavanirvāṇasvarūpe bhūcarīkhecaryau^59^ bhāvayet.

atra ca devīnām utpattyanantaraṃ svakuleśābhiṣeke^60^ sati svakuleśamudrā boddha[Ṅ fol. 262^v^]vyā.^61^

etā pañcadaśa yoginyaḥ ṣoḍaśābdāḥ sūryamaṇḍalasthā bhinnāñjanābhā^xli^ bodhicittasvabhāvā jvalitapiṅgalordhvakeśās^xlii†^tāluke^†^^xliii^vajrasattvasvabhāvacaturaṅgulakapāladhāriṇyaḥ^62^ śirasi ca pañcabuddhasvabhāvaśuṣkapañca- muṇḍāni^xliv^bibhratyo raktavartulatrinetrā daṃṣṭrākarālavadanāḥ pañca- daśamātṛkāsvabhāvaśuṣkapañcada-śamuṇḍamālālaṅkṛtā^63^^,xlv^vyāghracarmāvṛtakaṭinitambā^64^^,xlvi^ardhaparyaṅkanāṭyasthāḥ śavārūḍhāḥ pañcamudrādharāḥ. tatra—

akṣobhyaś cakrirūpeṇāmitābhaḥ^65^ kuṇḍalātmakaḥ |

ratneśaḥ kaṇṭhamālāyāṃ haste vairocanaḥ sthitaḥ^xlvii^ || 1 ||

mekhalāyāṃ sthito ’moghaḥ sarvāṅge vajradhṛk tathā |^xlviii^

gurvācāryeṣṭadevasya^66^ namanāya śirasi cakrikā || 2 ||

durbhāṣasyāśravaṇāya^67^ guror vajradharasya ca |

karṇayoḥ kuṇḍalaṃ dhāryaṃ^68^ mantrajāpāya kaṇṭhikā || 3 ||

mekhalā bhajituṃ mudrāṃ tyaktuṃ prāṇivadhaṃ rucakaḥ |^xlix^

keyūranūpuradharāḥ^69^^,l^ kṛṣṇāṅgo maitracittataḥ || 4 ||^li^

keśānāṃ raktapiṅgatā mahārāgatākhyāpanāya, krodhapratipādanāyordhvatā.^lii^kāyavākcetasām atirāgasvabhāvatvāt tatsvabhāvānāṃ^70^ netrāṇām mahārak- tatā.^71^^,liii^ bhavanirvāṇasvabhāvau bāhū. mānādidoṣān kartituṃ kartikā.^liv^ traidhātukaviśuddhyā skandhādicaturmārarudhirapūrṇaṃ^lv^ trikhaṇḍaṃ^72^ sakalavi [Ṅ fol. 263^r^]kalpaśarīri^73^^,lvi^ kapālam. dharmasambhoganirmāṇaviśuddhyā tribhaṅgam.^74^^,lvii^ svābhāvikakāyaviśuddhyā^lviii^ śarīrayaṣṭiḥ. anāvaraṇatākhyāpanāya vyāghracarmavasanatā. traidhātukānālambanatākhyāpanāyā lambanaikacaraṇatā.^75^^,lix^ ekaras-atākhyāpanāyaikapādākrāntabhūtalatā.

tadanantaraṃ hṛdvartibījavinirgataiḥ^76^ pañcākāraraśmibhir akaniṣṭhabhuvanavartijñānasattvasvabhāvaṃ nairātmyācakram ānīya hṛdbīje praveśayet. jñānasattvasamayasattvayor aikyaṃ kṛtvā^77^ nairātmyāhaṃkāram udvahan nairātmyāsamo^78^^,lx^ bhavet.

atra ca ṣaḍaṅgayogavyavasthārtham anukrameṇa kṛṣṇaraktapītaharitanīlaśuklavarṇā^79^ bhāvanīyāḥ.^lxi^

tatra bhāvanāprakarṣaprakrameṇa prathamaṃ meghasaṃchannapūrṇacandravad^80^ bhāti. tato ’pi prakarṣān māyāvad bhāti. tato ’pi prakarṣāt svapnavat prakāśate. tadanantaraṃ prakarṣaparipākāt svapnajāgraddaśayor abhedaprāpto mahāmudrāyogī sidhyati,^lxii^ ity utpattikramaḥ.

anyatra^lxiii^ bolakakkolasaṃyogān mahāsukharūpi paramaviramamadhyagaṃ^81^^,lxiv^ bodhicittaṃ jāyate yat tad eva pañcadaśakalātmakaṃ jhaṭiti pūrvoktavarṇacihnas-aṃsthānapañcadaśayoginīrūpaṃ^82^ paśyet,^lxv^ tasyāpi^83^ pañcaskandhacaturdhātuṣaḍviṣ-ayakāyavākcittasvabhāvatvād iti [Ṅ fol. 263^v^] gambhīrotpattikramaḥ.^lxvi^

jhagiti bījam anavalokayann^84^ eva pañcadaśayoginyātmakaṃ maṇḍalacakraṃ paśyed iti utpannakramaḥ.^lxvii^

atha pariniṣpannakramaḥ. vajraśarīre khalu jñānādhiṣṭhite^lxviii^ dvātriṃśan^85^ nāḍyo mahāsukhasthānāt sravanti. tāś ca pañcadaśa yoginya iti śarīram eva nairātmyācakrātmakam. tathā hi lalanārasane kaṇṭhād^86^ ārabhya nābhiṃ yāvad vāmetarapārśvavar-tinyau candrasūryākhye. nābher adhas te eva yonināḍyau lalanārasane akṣobhyarudhiravahe.^lxix^ avadhūtī śiraḥkaṇṭhahṛnnābhiyonimadhyasthā^87^ bodhicittavahā.^88^ etā nāḍyo nairātmyā.

abhedyāsūkṣmeśiraḥśikhāsthe yathāsaṃkhyaṃ nakhadantakeśaromalakṣaṇayugmayugalavahe^89^ vajrā. divyā dakṣiṇakarṇe tvaṅmalavahā,^90^ vāmā pṛṣṭhavaṃśe piśitavahā gaurī. vāmanīkūrmaje vāmakarṇabhrūmadhyasthe^91^ snāyvasthimālāvahe^lxx^ vārī. bhāvakīseke cakṣurbāhumūlasthe^92^ vṛkkahṛdayavahe^lxxi^ ḍākinī.

doṣāvatīmahāviṣṭe kakṣastanavartinyau cakṣuḥpittavahe pukkasī. mātarāsarvaryau nābhināsāgrasthe^93^ phupphusāntramālāvahe^94^^,lxxii^ śabarī. śītadoṣme mukhakaṇṭhasthe pārśvatantūdaravahe caṇḍālī. pravaṇā hṛdaye viṣṭhāvahā, hṛṣṭavadanā^lxxiii^ liṅge^95^ sīmantamadhyagā ḍombī.^lxxiv^

svarū[Ṅ fol. 264^r^]piṇīsāmānye^lxxv^ meḍhragudayoḥ^96^ śleṣmapittavahe gaurī. hetudāyikāviyoge ūrujaṅghayoḥ śoṇitasvedavahe caurī. premaṇīsiddhe pādāṅguṣṭhapādapṛṣṭhayor^97^ medaḥkhedāśruvahe^98^^,^^lxxvi^ vettālī. pāvakīsumane^99^ aṅguṣṭhajānudvayasthe. tatra pūrvā kheṭavahā, aparā bālasiṃhāṇavahā.^100^^,lxxvii^ te ime ghasmarī.

hṛtkamalakarṇikāpūrvādidaleṣu yathākramaṃ trivṛttā-kāminī-gehā-caṇḍikā-māradārikāḥ. tatra prathamaṃ^101^ nāḍīdvayaṃ bhūcarī, [śeṣāḥ khecarī.]^102^ atra ca yā nāḍī yaṃ prasūte^103^ puṣṇāti gacchati vā sā tadvahā.^lxxviii^

kiṃ caitāḥ^104^ kāyavākcittadharmasambhoganirmāṇatribhavasvabhāvāś^105^catuścakreṣu śarīreṣu vyavasthitāḥ.^lxxix^ tatra nirmāṇacakraṃ viśvavarṇa- catuḥṣaṣṭidalaṃ^106^^,lxxx^ kṛṣṇa-a-kārabījaṃ^107^ nābher adho vyavasthitam ūrdhvamukhaṃ ca. dharmacakraṃ śuklāṣṭadalakamalaṃ^lxxxi^ kṛṣṇavarṇa-hūṁ-kārabījaṃ hṛddeśe vyavasthitam.^108^ kaṇṭhe sambhogacakraṃ raktaṣoḍaśadalaṃ^109^ raktapraṇavabījam. śirasi śukladvātriṃśaddalaṃ śukla-haṃ-kārabījam adhomukhaṃ kṣaratpīyūṣadhāraṃ mahāsukhacakram. atrānandakṣaṇabhedādivyavasthā gurūpadeśato boddhavyā.^110^

anābhogayuganaddhādvayavāhi^111^ bodhicittasākṣātkaraṇaṃ^lxxxii^ svābhāvikaḥ kramaḥ.

tato bhāvanākhinno nairātmyāhaṃkāram u[Ṅ fol. 264^v^]dvahan mantraṃ^112^ japet. tatrāmī sahajasiddhāḥ praṇavādyāḥ svāhāntāḥ pañcadaśasvarasvabhāvā a-kārādayo mantraḥ, tadyathā—oṁ a ā i ī u ū ṛ ṝ ḷ ḹ e ai o au aṁ svāhā.^lxxxiii^

raktanairātmyāhaṃkāram^113^ udvahan [purakṣobhamantraṃ]^114^ japet. tatrāyaṃ mantraḥ—oṁ a ka ca ṭa ta pa ya śa svāhā.

oṁ akāro mukhaṃ sarvadharmāṇām ādyanutpannatvāt oṁ āḥ hūṁ phaṭ svāhā—balimantraḥ.

oṁ āḥ hūṁ—samayādhiṣṭhānamantraḥ.

tadanantaraṃ maṇḍalacakrasākṣātkāradaśāyāṃ^115^ stanau hṛtvā svābhāṅganākakko- lamadhyavarti^116^^,lxxxiv^ bolaṃ kuryāt. pārśvadvayaṃ ghaṇṭāṃ vidadhyāt.

[mantrayāna]^117^śāstrasāraṃ^118^^,lxxxv^ jñātvā yatnena sadguroḥ |^lxxxvi^

kṛpayā vihito ’smābhir nairātmyāyāḥ prakāśakaḥ ||

gahanamaṇḍalacakraviniścayo

bata bhavet katham atra śarīriṇām |

śabaranāthapadāmbujareṇubhir

yadi na rūkṣitamastakatā^119^ bhavet ||^lxxxvii^

[abhisamayasuvistṛtau]^120^ yad āptaṃ

kuśalam anena bhavet^121^ samastalokaḥ |

kuliśadharapadapratiṣṭhitātmā^122^

hatabhuvanatrayaduḥkhadaurmanasyaḥ^123^ ||^lxxxviii^

nairātmyāprakāśaḥ samāptaḥ.

|| kṛtir iyaṃ śrīmatpaṇḍitācāryāvadhūtādvayavajrapādānām iti ||

## Notes

^I^ The opening *oṁ namaḥ śrīnairātmyāyai* is a scribal homage.

^ii^ This verse, in Mālinī metre, serves as a *maṅgalācaraṇa* in other texts attributed to Advayavajra. We find it in Mar pa Chos kyi dbang phyug’s Tibetan translation of the *Saptākṣarasādhana*:

*kun du rtog pa yongs su spangs pa’i chos skur gang gsungs dang ||*

*dpe med bde ba rtsal gyis mdzes pa longs spyod rdzogs sku dang ||*

*gang gi gnas la phan par mdzad pa las ni sprul pa’i sku ||*

*bcom ldan rdo rje sems dpa’ de yis khyed la bde legs shog ||* (D fol. 130r–v) (read *rtsal* as *tsam*).

It is absent from this text as printed in Bhattacharyya’s edition of the *Sādhanamālā*.

The verse is also found at the beginning of Advayavajra’s *Cakrasaṃvaropadeśa, for which rMa Chos ’bar’s translation reads:

*gang zhig kun du brtags pa yongs su spangs pa’i chos sku dang ||*

*dpe med bde ba tsam gyis mdzes pa’i longs spyod rdzogs sku dang ||*

*gang gi thugs rje sa rnams phan mdzad sprul pa’i sku brjod pa ||*

*bcom ldan rdo rje sems dpa’ de yis khyod la bde legs shog ||* (D fol. 139r)

A rather free rendering is found in the closely related *Cakrasaṃvarasādhana, originally translated by Ba reg thos dga’ and Vajrapāṇi but later revised:

*gang zhig rnam dag kun du rtog yangs mkha’ ’dra’i chos sku dang ||*

*dpe med phun tshogs bde bas rnam mdzes longs spyod rdzogs sku nyid ||*

*sa rnams kun du phan mdzad rab gsal sprul pa’i skur brjod gang ||*

*bcom ldan rdo rje sems dpa’ de yis khyed la bde legs shog ||* (D fol. 133v)

The verse is also transmitted in the so-called *Sādhanavidhāna* codex, on fol. 3r, in an *adhyātmahomavidhi*. Péter-Dániel Szántó (personal communication) surmises that the colophon to this brief text is written in old Newar and amounts to saying that the *vidhi* was extracted from a *ṭippanī* on the *Saṃvarodayatantra*. Here the manuscript reads: *parahitaparikalpaṃ dharmmakāyajam āhu* | *nirupamasukhapātraṃ cārusabhogakāyaḥ* | *bhuvanahitavināt yasya nirmmāṇakāyaḥ* | *sa bhavatu bhagavān vaḥ śreyase vajrasatvaḥ* ||

Harunaga Isaacson (personal communication) has also read the verse in manuscripts of the *Yogāmbaratantra* and the (proto-)*Kalparājatantra*, both Nepalese compilations. It would thus appear that the verse achieved some popularity in Nepal. The NGMPP online catalogue offers a transcription of the verses in NAK 4/2917 (A 142-12), a witness of the *Yogāmbaratantra*. According to this transcription, the verse also reads *°pātraṃ* for *°mātraṃ*, *nirmāṇakāya* for *nirmāṇakāyaṃ*, and *sa bhavatu bhagavān* for *bhavatu sa bhagavān*.

The reading *pātra* can probably be discarded given the Tibetan evidence, but it is an interesting variant. The readings *sa bhavatu* and *bhavatu sa* are effectively indistinguishable. One may wish to read *nirmāṇakāyaḥ* in the third *pāda*, in which case the meaning is not that Vajrasattva is taught *to be* the *nirmāṇakāya* (because of his accomplishing what is beneficial for the world), but that he simply *has* the *nirmāṇakāya* (while he is the first two *kāya*s). Some further research into Advayavajra’s position on the three *kāya*s may reveal which of these understandings is more appropriate. On the reading *nirmāṇakāyaḥ*, the relative pronoun *yasya* is, at least for my personal taste, slightly smoother.

At this point it may be worth comparing the above three Tibetan translations of this verse with mTshur Ye shes ’byung gnas’s effort here for the NaiPra:

*kun du rtog pa yongs spangs chos skur gang brjod pa ||*

*dpe med bde ba tsam mdzes longs spyod rdzogs pa’i sku ||*

*sa yi phan pa’i rgyur gyur gang gi sprul pa’i sku ||*

*bcom ldan rdo rje sems dpa’ khyod des dge bar shog ||*

The translation *rgyur gyur* for *vidhānāt* is difficult to account for; it also appears to be an adjective qualifying either *gang* or *sprul pa’i sku* rather than an ablative form. It is perhaps Chos kyi dbang phyug’s translation that has the clearest rendering of this *pāda* with *gang gi … mdzad pa las*. Similarly, the syntax of the final line, with *rdo rje sems dpa’* separated from the pronoun *de*, is considerably more opaque than the other translations. Of the four Tibetan translations, the two by rMa Chos ’bar and Ba reg thos dga’ appear to syntactically construe *nirmāṇakāya* with *√ah* and hence lend support to reading *°kāyaṃ* instead of *°kāyaḥ*. The remaining two Tibetan translations are less clear on this matter.

Although it may be entirely coincidental, it is nonetheless noteworthy that the opening verse of Ratnākaraśānti’s *Bhramaharasādhana* is also in Mālinī metre.

^iii^ The manuscript reads as follows for the first half of this verse: *ekatra vitataṃ spaṣṭaṃ mabodha((ṃ)) laghuvistaram* (the *anusvāra* is doubtful). Several conjectures may be considered, though none seems highly certain. One possibility is to read *abodhalaghuvistaram* in the second *pāda*, interpreting the first verse half as: ‘[A *sādhana* which is] clearly (*spaṣṭa*—taken as an adverb, adjective, or both) laid out in a single place, with a small amount of prolixity for those who lack understanding.’ Another option is *saṃbodhalaghuvistaraṃ*, interpreting the compound as ‘with a small amount of prolixity for the sake of understanding’. The *Dhīḥ* edition reads *abodhaṃ laghuvistaram* without any variants reported and glosses *abodhaṃ* in Hindi as *samajh meṃ na āne vālī* (‘not coming within understanding’), which seems improbable as way for Advayavajra to describe his *sādhana*. T_D_ reflects the first or last possibility, though its intended meaning is unclear: *gcig tu gsal la rgyas pa yis || mi rtog nyung la rgyas pa dag ||*

My tentative conjecture is *sabodhaṃ laghuvistaraṃ*, as it feels like a natural reading and is graphically close to the transmitted reading of *mabodhaṃ*. The reading *sabodhalaghuvistaraṃ* can also be considered, either as a *viśeṣaṇasamāsa* or with another interpretation. The entire verse as printed can be translated as follows: ‘In accordance with my understanding (*yathāmati*) and with scripture (*yathāgama*), I will teach a *sādhana* of Nairātmyā, clearly laid out in one place with understanding and with a small amount of detail (*vistara*).’

^iv^ To illustrate some of the differences between the present edition and that published in the *Dhīḥ* journal, I briefly note the following for the clause starting with *yogī* and ending with *utpādya*: The *Dhīḥ* edition retains the reading *śmaśānādi°*; it does not report the manuscript’s reading of *sukhāsanopaviṣṭaṣṭo*; it reads *niḥsakto* where the manuscript (correctly) reads *niḥsaṅgo*; it retains the manuscript’s reading of *niḥśaṅka*, which it prints in compound. There are many such differences throughout the two editions, but I will not dwell on them further.

^v^ Where the Sanskrit manuscript reads *tatas thadīyai*, T_D_ reads *de nas de’i gsal ba’i*, suggesting the conjecture *tatas taddīptai*. The reading *tatas tadīyai*, however, reads well and requires minimal emendation, as *stha* and *sta* are similar in this scribe’s hand.

^vi^ T_D_ reflects a plural form of *akaniṣṭhabhuvanavartin*: *’og min gyi gnas na bzhugs pa rnams*. Given that what follows is a description of only the eight-faced Heruka, the plural form can be regarded as an error.

T_D_ also places *’og min gyi gnas na bzhugs pa rnams* before *ākṛṣya* (*bkug ste*), which may be simply for syntactical naturalness in Tibetan rather than a reflection of a different reading in Sanskrit.

^vii^ T_D_ lacks a reflex of *avadāta* within the compound *indukundāvadātadakṣiṇamukha*: *g.yas pa’i zhal ni zla ba dang | kun da lta bu’o ||*

^viii^ The conjecture *alimalinetarasakalavadanaṃ* where the manuscript reads *alamalinetara°* is compelling in view of HeTa 2.5.11cd–12: *mūlamakhaṃ mahākṛṣṇaṃ dakṣiṇaṃ kundasannibham || vāmaṃ raktaṃ mahābhīmam ūrdhvāsyaṃ vikarālinam | caturviṃśatinetrāḍhyaṃ śeṣāsyā bhṛṅgasannibhāḥ ||* T_D_ suggests reading *atimali°* with *shin tu gnag pa*, which has the advantage of paralleling the preceding compounds.

^ix^ Here the transmitted text has suffered from what was likely a scribe’s eye-skip. I offer this conjectural reading based on T_D_ as well as similar formulations in two other *sādhana*s by Advayavajra. First, the *Saptākṣarasādhana* reads: *… yathāvidhinā pūjayet vandayet | tatas teṣāṃ purataḥ pāpadeśanāpāpākaraṇasaṃvaraṃ puṇyānumodanātriśaraṇagamanabodhicittotpāda-ātmabhāvaniryyātanā-adhyeṣaṇāyācanāś ca kṛtvā …* (ed. p. 460; *sandhi*, orthography, etc. as per the edition). In the NaiPra, the Tibetan translation indicates the inclusion of *puṇyapariṇāmanā*, but the word *yācanā* is reflected neither in the Tibetan nor in the Sanskrit manuscript, which has resumed at the place where one expects to see it. Otherwise the two texts are evidently closely parallel.

Advayavajra’s *Vajravārāhīsādhana* reads as follows: *tadagrataḥ pāpadeśanā-pāpākaraṇasaṃvarapuṇyānumodanāpuṇyapariṇāmanātriśaraṇagamanabodhicittotpādādikaṃ kṛtvā …* (ed-f p. 59; ed-b p. 424) (*°pāpākaraṇasaṃvara°*] ed-f; *deest* in ed-b but recorded as a variant). Here *vandana* is not present, and *ātmabhāvaniryātana* and so forth have probably been replaced with the word *ādika*.

In the NaiPra, the *ca* before *kṛtvā* indicates that more than one word precedes, as in the *Saptākṣarasādhana*. I assume *pāpadeśanāpāpākaraṇasaṃvaraṃ* can be understood as a *samāhāradvandva*, but I can find no other attestation of the compound apart from in the *Saptākṣarasādhana*. The segmentation of the compounds might be explained because *pāpadeśanā* etc. represent actions related to dispelling hindrances, while *puṇyānumodanā* and so forth are related to accumulating merit. English (2002, pp. 122–24) describes various preliminary stages in similar *sādhana*s.

As for the first sentence (*atra ca prajñopāyayoḥ* etc.), I can find no parallel in other *sādhana*s, so I only rely on T_D_ for the proposed conjecture. If I have understood the Tibetan correctly, I believe Advayavajra is offering a justification for worshipping the eight-faced Heruka, i.e. Picuvajra, at the beginning of the *sādhana*: to put it somewhat baldly, a *sādhana* that includes worship (and meditation on) both male and female deities aids one in realising that insight and means have an identical nature.

The *Dhīḥ* edition offers a largely similar conjecture: *atra ca prajñopāyayos tādātmādhigamārthaṃ picuvajrapūjanam | tataḥ vandanāṃ pāpadeśanāṃ pāpākaraṇasaṃvaraṃ puṇyānumodanāṃ puṇyapariṇāmanāṃ triśaraṇagamanaṃ bodhicit-totpādātmabhāvaniryātanām adhyeṣaṇāñ ca* (p. 116). The editors may have either misread or silently conjectured singular accusative endings for *ātmabhāvaniryātanā* and *adhyeṣaṇā*, while leaving *bodhicittotpāda* in compound, which doesn’t seem justifiable. Given the parallel evidence, larger compounds seem more plausible.

^x^ Regarding the compound *śūnyatājñānavajrasvabhāvātmaka*, traditional authorities have interpreted *vajra* either as co-referential with *jñāna* or with *svabhāva*. The former interpretation is offered by, for example, Śākyarakṣita in his *Abhisamayamañjarī*, and the latter by Abhayākaragupta in ch. 4 of his *Abhayapaddhati* (English, 2002, 239–40 n. 273, n. 277; Isaacson, 2007, p. 292; Yang, 2014, pp. 140, 234).

^xi^ T_D_ renders *śūnyatājñānavajrasvabhāvātmakāḥ sarvadharmāḥ* in Tibetan, indicating that, perhaps, these words were not understood as part of the mantra: *dngos po mtha’ dag gi de kho na nyid kyi dngos po bsdus pa | oṁ shū nya tā dznyā na badzra sva bhā ba ātma ko ’ham | stong pa nyid kyi ye shes rdo rje rang bzhin gyi bdag nyid la chos thams cad ces bya ba’i sngags kyi don bsgoms pas rab tu mi gnas pa’i ngo bor gnas so ||* Indeed the mantra *oṁ śūnyatājñānavajrasvabhāvātmako ’ham* generally stands on its own; however, it would appear that Advayavajra is fond of this reformulation of the mantra, which emphasizes the emptiness of all phenomena (see English, 2002, 128).

Evidence to support this can be found in other *sādhana*s composed by Advayavajra, such as the *Saptākṣarasādhana*: *tataḥ oṁ śūnyatājñānavajasvabhāvātmakāḥ sarvadhāḥ oṁ śūnyatājñānavajasvabhāvātmako ’ham iti sakala-vastutattvasārasaṃgrāhakaṃ mantrārtham āmukhīkurvvan …* (ed. p. 460). Note, however, that Mar pa Chos kyi dbang phyug’s Tibetan translation of the *Saptākṣarasādhana* does not reflect the first *oṁ* and evidently represents the words as a stand-alone clause: *de nas chos thams cad ni stong pa nyid kyi ye shes kyi rdo rje’i bdag nyid de | oṁ shū nya tā dznyā na badzra sva bhā ba ātma ko ’ham/ zhes bya ba dngos po ma lus pa’i de kho na nyid sdud par byed pa’i sngags kyi de kho na mngon du byed cing |* (*de kho na* may be a corruption of *don* or *don kho na*) (D fol. 131r).

Advayavajra’s *Hevajraviśuddhinidhi* also has a formulation resembling the *Saptākṣarasādhana*: *etadantaraṃ sarvadharmapravicayalakṣaṇayā prajñayā sarvadharmān pratītyasamutpādakān svabhāvānutpannān adhimuñcan, tadarthadyotakatvāt sakalavastutattvasārasaṅgrāhakatvena ca,* oṁ śūnyatājñānavajrasvabhāvātmakāḥ sarvadharmmāḥ | oṁ śūnyatājñānavajrasvabhāvātmako ’ham* iti mantram imaṃ manasā paṭhitvā* … (*tadartha*°] em.; *tadarthaṃ* ms) (ms fol. 66r7–v2). Here ’Gos gZhon nu dpal’s Tibetan translation completely lacks any reflex of the words in question (Dg fol. 176r). In the second, anonymous translation, the translator treats the passage as follows: *de ltar bla na med pa’i chos thams cad rab tu ’byed pa’i mtshan nyid kyi shes rab kyis chos thams cad rten ’brel las skyes pa tsam rang bzhin gyis gzod ma nas skye ba med par gsal bar shes par bya ste | dngos po ma lus pa’i bde ba de kho na nyid kyi snying por bsdus pa’i stong pa’i ye shes kyi rdo rje’i rang bzhin gyi chos shes nas oṁ shū nya tā dznyā na badzra sva bhā va ā tma ko ’ham zhes pa’i sngags de yid kyis bzlas te |* (Da fol. 163r).

Finally, the extended mantra is also found in some recensions of the *Vajravārāhīsādhana*. Finot’s edition reads: *tataḥ, oṃ śūnyatājñānasvabhāvātmakāḥ sarvvadharmmāḥ, oṃ śūnyatājñānavajrasvabhāvātmako ’ham iti mantrārtham āmukhīkurvan muhūrttam apratiṣṭharūpena tiṣṭhet* (perhaps *vajra* is missing from the first *śūnyatājñāna°*) (ed-f p. 59). Bhattacharyya’s edition in the *Sādhanamālā* (ed-b p. 424) lacks the first half of the mantra, as does the translation by mTshur ston dBang gi rdo rje (Dt fol. 181r). Yar lung Grags pa rgyal mtshan’s translation of the same text, however, does reflect something of the longer mantra: *de nas chos thams cad rdo rje’i rang bzhin gyi stong pa nyid du byas nas | oṁ shū nya tā dznyā na badzra svabhā ba ātma ko ’ham | zhes pa’i sngags kyi don mngon du byas nas skad cig gis mi gnas pa’i skur bsam par bya’o ||* (Dy fol. 229v6–7)

Taken altogether, this evidence affirms that Advayavajra had a special preference for an ‘enhanced’ formulation of the popular mantra, which may have caused some confusion for Tibetan translators. One lingering doubt is that the other attestations of the mantra appear to put *sarvadharmāḥ* first and *aham* second, whereas the NaiPra does the opposite. Given that T_D_ and the Sanskrit witness both support this seemingly reversed order in the NaiPra, I do not suggest emending; however, there is some doubt as to whether it is correct.

One further note should be made about the conjecture *sakalavastutattvasāra-saṅgrāhakaṃ* where the manuscript reads °*saṅgrahākātmakaṃ*. Alternative conjectures could be °*saṅgrāhātmakaṃ* or the more minimal °*saṅgrāhakātmakaṃ*, but in the latter case a noun rather than an adjective before *ātmaka* would be preferable. T_D_ shows no reflex of *ātmaka*: *dngos po mtha' dag gi de kho na nyid kyi dngos po bsdus pa*. The term, with slight variants, regularly describes and even serves as a name for the *mantra* taught in the passage at hand: Saroruhavajra, for example, introduces the *mantra* as *niḥśeṣavastutattvasārasaṅgrāhaka* (p. 101). Given the current conjecture, it is notable that here the term does not in fact describe the *mantra* but rather the *mantrārtha*, but this is also the case in Advayavajra's *Saptākṣarasādhana* (assuming the readings are correct): *sakalavastutattvasārasaṅgrāhakaṃ mantrārtham āmukhīkurvan* (p. 460).

^xii^ For *tataḥ praṇidhim anusmṛtya, samādher vyutthāya*, T_D_ lacks a reflex of *samādher* and renders *praṇidhim anusmṛtya* freely: *de nas smon lam gyis dran pas langs te |*

^xiii^ One may consider accepting the manuscript’s reading of *aṇusaṃhater vvajraiḥ*, understanding it in the sense of ‘vajras from an assemblage/coming together of atoms’, but the mention of atoms does not suit very well the context. Other possible readings are *aṇusaṃhatair vajraiḥ* and *aṃśusaṃhatair vajraiḥ*, but these options make it so that it is the vajras themselves that are joined together (*saṃhata*) by means atoms or light; rather, the idea is probably that the rays or atoms are tightly joined (*saṃhata*) and thus form vajras.

The Tibetan translation reads *’od zer gyi rdul phra rab kyi tshogs ’phros pa des*, thus replacing the vajras with light rays and maintaining the atoms (or minute particles). This renders the atoms less out of context, but it is at odds with the expected procedure.

I suggest reading *aṃśusaṃhater vajraiḥ* partially on the basis of Ratnākaraśānti’s MuĀ, commenting on HeTa 1.3.3:

*purastād agnivarṇena repheṇa sūryamaṇḍalaṃ dhyātvā tanmadhye hūṃkāreṇa viśvavajraṃ vicintya tatkiraṇasūkṣmavajraiḥ sphuradbhiś caturdiggatair atyantaṃ ghanībhāvāt prākāraṃ bhāvayet* (as cited in Isaacson, 2007, p. 293).

We see that it is indeed additional vajras from the initial vajra that produce the fence, and these are described as ‘[made of] the light rays of that [initial vajra]’. Since the characters for *ṇu* and *śu* can be extremely similar, I regard the proposed conjecture as a relatively minor intervention.

^xiv^ The compound *pañjarabandhana* can be understood as a *dvandva*. This section of the *sādhana* derrives from HeTa 1.3.3, where the form *pañjarabandhanaṃ* (or *pañjara bandhanaṃ*) appears to be *metri causa*: *repheṇa sūryaṃ purato vibhāvya tasmin ravau hūṁbhavaviśvavajram | tenaiva vajreṇa vibhāvayec ca prākārakaṃ pañjarabandhanaṃ ca ||*

^xv^ For the final sentence of this paragraph—*raviviśvavajrābhyāṃ ca raśmībhūya samantataḥ prasṛtābhyāṃ tat sarvaṃ dṛḍhīkuryāt*—T_D_ yields a different sense, which is probably the result of either free translation or misunderstanding: *nyi ma dang sna tshogs rdo rje dag las ’od zer dpag tu med pa byung bas de ltar de dag thams cad brtan par bya’o ||* (‘Thus one should make all of this stable by infinite light emerging from the sun and the crossed vajra’). The Sanskrit can be translated as follows: ‘One should make all of this stable by the sun and crossed vajra which, turning into light, spread everywhere.’

^xvi^ The manuscript’s reading of simply *ūrdhvāṃ* in describing the *prajñā* is not impossible, but it is telegraphic and may even suggest the opposite configuration—namely, with the tip of the triangle pointing upwards. With *steng yangs pa*, T_D_ indeed suggests reading something like *ūrdhvaviśālā*; however, other possibilities, such as *upari viśālā* or *ūrdhvavistarā*, cannot be entirely ruled out. The compound *ūrdhvaviśāla* is relatively rare, but it has at least one attestation describing the *dharmodaya/ā*, in Umāpatideva’s *Vajravārāhīsādhana* (verse 16, ed. p. 236), where it is incorporated into a verse in Upajāti metre.

^xvii^ T_D_ renders *tadantarvarti* of the previous sentence and *tanmadhye* here as *de’i steng du*. It renders *madhyavarti* in the following compound as *la gnas pa*. The translator thus appears to have made a conscious decision to avoid translating these with words meaning ‘inside’ or ‘in the middle of’. Elsewhere in the translation, *madhya* is generally rendered as *dbus*.

^xviii^ The word *maṇḍala* (or a synonym) appears to have dropped out of the manuscript, again perhaps due to an eyeskip. Strangely, the word is also not reflected in T_D_: *sna tshogs rdo rje’i dbus su yang rlung dang | me dang | chu dang | sa rnams ni du ba dang | dmar po dang | dkar po dang | ljang gu rnams | gzhu dang | zur gsum pa dang | zlum po dang | gru bzhi rnams | yaṃ raṃ baṃ laṃ rnams yongs su gyur pa las steng nas steng du blta’o ||*

^xix^ T_D_ lacks a reflex of *ākāra* at the end of this compound: *… zlum po dang | gru bzhi rnams |*

^xx^ T_D_ reads *rta babs bzhi pa* after *caturdvāram*, reflecting *catustoraṇam*. This word is certainly fitting, but at present I feel it is impossible to say whether it was added to the translation or lost from the Sanskrit witness.

Here the text verges on entering *anuṣṭubh*, as it is inspired by verses that can be traced back to at least the *Sarvatathāgatatattvasaṅgraha*, and which are quoted or employed with variations in countless texts (see Tribe, 2016, 143 n. 24 for references, and pp. 254–5 for Vilāsavajra’s version of these in the *Nāmamantrārthāvalokinī*; see also, for example, HeTa 1.10.21).

^xxi^ T_D_ lacks a reflex of *atra*. Perhaps *tatra* with a partitive sense would read better here, but I don’t see very strong reasons to discount *atra*.

^xxii^ T_D_ treats the words for each of the trees in the eight charnel grounds as if they were nominative forms. Ṅ has two instances of the words without case endings, one instance with a form that is corrupt in another way, and five instances of locative forms. If we were to accept nominative forms, a conjunction such as ‘*ca*’ would also be natural; I therefore find this possibility unlikely, and adopting it would require major alterations to the transmitted text. Compounded forms such as *harītakīvṛkṣamecakavarṇaḥ* can, I believe, also be discounted, as they appear unprecedented and unnecessary. If locative forms were intended, we can account for the error in the Sanskrit manuscript given that *kṣa* and *kṣe* are so graphically similar; we also see that forms less susceptible to confusion (e.g. *śākhini* or *tarau*) are here unambiguously locative.

According to some other accounts of the eight charnel grounds, there is a *śirīṣa* in the east (see Gerloff, 2020, vols. 2, pp. 739–740; English, 2002, p. 140). Like the NaiPra, however, the anonymous *Aṣṭaśmaśāna places a *harītakīvṛkṣa* in the east. The Pandanus Database of Plants identifies *śirīṣa* as *Acacia lebbeck Willd.* (Siris tree) and *harītakī* as *Terminalia chebula Retz.* (Chebulic myrobalan). It is unclear to me if Advayavajra regarded the two as synonyms.

^xxiii^ These colours should be understood as qualifying the *yakṣa*s (also called *maharddhika*s or *kṣetrapāla*s in some sources) and not the *dikpati*s, whose colours are described later as being ‘as they are commonly known’. The colours given by Advayavajra throughout this passage do not agree with the colours of the *yakṣa*s given in the *Aṣṭaśmaśāna. The *Aṣṭaśmaśāna, for instance, specifies that the first *yakṣa* is white (*śukla*) instead of dark (*mecaka*). Here and below in the list of cloud colours T_D_ renders *mecakavarṇa* as *ser po* (yellow).

^xxiv^ The structure of the formulation changes with the third *śmaśāna*, which is also reflected in T_D_. Other accounts of the eight charnel grounds, including visual depictions, make it clear that it is the *yakṣa*s who are *in* the trees, whereas the *dikpāla*s are nearby the trees—for example, the *Aṣṭaśmaśāna reads:

*tatrāśokavṛkṣe maharddhiko jvalākulakaraṅko nāma makaramukho raktaḥ | vṛkṣādhasi dikpatir varuṇo nāgāsanaḥ śuklaḥ |* (ms. 4v; orthography adjusted; MS reads *nāmo* and *vāruṇo*; note the variant colours)

The NaiPra, although more telegraphic, should probably be understood accordingly.

^xxv^ Other sources name the tree here *parkaṭī* (Gerloff, 2020, vol. 2, p. 740), for which *Amarakośa* 4.32 gives *jaṭī* as a synonym.

^xxvi^ *Amarakośa* 1.69 records *naravāhana* as a name for Kubera, but here it should be understood as referring to Nairṛti/Nirṛti, who indeed generally has a human as his mount (Wessels-Mevissen, 2001, 98 ff.). The Tibetan translation appears to have mistranslated this sentence, perhaps being thrown off by the direction being mentioned at the end: *bden bral du la da dza ba ḍu’i shing dang | mi’i gdong can dang | bden bral dkar po’o ||* (*du la da dza ka ba ṭu’i*] T_P_ T_N_; *dul da dza ba ḍu’i* T_D_).

^xxvii^ T_D_ renders *bhūteśa* as *’byung po* where one expects *’byung po’i dbang po*. It also construes *citra* as qualifying *nyagrodhapādapa* and adds a finite verb: *dbang ldan gyi mtshams su ’byung po dang | khyu mchog gi gdong can no || nya gro dha sna tshogs pa bsgom mo ||*

^xxviii^ As mentioned above, T_D_ renders *mecaka* as *ser po*. For *śukla* T_D_ reads *skya bo*, whereas elsewhere in the translation *śukla* is rendered as *dkar po*. The Sanskrit *pāṇḍu* is rendered well here as *dkar ser*, but above it had been rendered simply as *dkar po*. The colours Advayavajra gives for the clouds match exactly those given in the *Aṣṭaśmaśāna.

^xxix^ The word *cintanīyāḥ* has no reflex in T_D_.

^xxx^ Here again it is evident that the transmitted text, which reads *evaṃ dikpālāś cāṣṭau yakṣavarṇṇāni bodhavyāni*, has suffered from another eye-skip. The neuter form *°varṇāni* lends support to what can be understood from T_D_: namely, that there should be a second sentence regarding the colour of *caitya*s. The first sentence, which in T_D_ reads *de ltar phyogs skyong brgyad kyi kha dog ni grags par zad do*, is relatively unproblematic. Here I ‘back translate’ *grags par zad do* with *prasiddha*—attestations for this correspondence cannot be found in the translation of mTshur Ye shes ’byung gnas, but we do find it elsewhere, such as in ’Gos Lhas btsas’s translation of Ratnākaraśānti’s MuĀ ad HeTa 2.4.53 (ed-t p. 183; D fol. 186r).

The second sentence is slightly more problematic. T_D_’s reading—*mchod rten brgyad kyi kha dog kyang rtogs par bya’o*—suggests something along the lines of, *aṣṭacaityānāṃ varṇā api boddhavyāḥ*. However, our Sanskrit manuscript indicates that the sentence ends with *yakṣavarṇāni boddhavyāni*. The Tibetan text may have suffered from corruption or mistranslation, since, as it stands, it is not at all clear how one should understand the colours of the *caitya*s. At least in the *Hevajraprakāśa*, where the colours of the *caitya*s are mentioned, they indeed match the colours of the *yakṣa*s.

The *Dhīḥ* edition leaves the manuscript reading unedited, but notes the divergent Tibetan translation (*de ltar phyogs skyong brgyad kyi kha dog ni grags par zad do || mchod rten brgyad kyi kha dog kyang rtogs par bya’o*) and offers a (very faulty) Sanskrit rendering: *evaṃ dikpālāṣṭau varṇāni prasiddhāni | stūpāṣṭau varṇāni ca bodhavyāni |*

^xxxi^ T_D_ reads *dkyil ’khor gyi ’khor lo’i nang du* where the Sanskrit manuscript reads *maṇḍalamadhye*, suggesting the reading *maṇḍalacakramadhye*.

^xxxii^ T_D_ lacks a reflex of *tat* in *tatkamalamadhyapūrvādicaturdaleṣu*: *padma’i nang shar phyogs la sogs pa’i ’dab ma bzhi*. The compound should be understood as ‘in the middle of that lotus and on the petals beginning in the east’. By contrast, T_D_ suggests understanding ‘on the four petals inside the lotus beginning in the east’.

^xxxiii^ T_D_ erroneously reads *ro bco lnga dang ldan par* for *pañcadaśa śavān*.

^xxxiv^ The compound *candrasūryasabījakartikāpariṇāma* as rendered in T_D_ does not reflect *sabīja*: *zla ba dang | nyi ma dang | gri gug yongs su gyur pa las*.

^xxxv^ T_D_ lacks a reflex for *jñāna* in *samatājñānavān*: *mnyam pa nyid dang ldan pa*.

^xxxvi^ This last sentence is a paraphrase of HeTa 1.8.6c–7: *ādarśajñānavāṃś candraḥ samatāvān saptasaptikaḥ || bījaiś cihnaṃ svadevasya pratyavekṣaṇam ucyate | sarvair aikyam anuṣṭhānaṃ niṣpattiḥ śuddhadharmatā ||* Here I constitute these verses in accord with Kamalanātha’s commentary, the *Ratnāvalī*. It should be noted, however, that the manuscript witnesses of the tantra provide a number of variant readings and, based on these readings and other evidence, one may wish to constitute the verses slightly differently.

^xxxvii^ T_D_ freely renders this portion of the text, combining *dveṣātmikā* and *vijñānaskandhātmikā* into a single compound: *zhe sdang dang | rnam par shes pa’i phung po’i bdag nyid* …

^xxxviii^ T_D_ may reflect a different reading for *prajñopāyasvarūpā bahirupāyarūpa-khaṭvāṅgāliṅgitakandharā*: *thabs dang shes rab kyi ngo bo’i gzugs kha tvaṃ gas gzugs ’khyud pa’o*. The translation *ngo bo’i gzugs* may represent something different for *°svarūpā*, there is no reflex of *bahirupāya°*, and the second *gzugs* may be based on a Sanskrit word other than *kandhara*, which the translator previously rendered as *mgul*.

^xxxix^ T_D_ erroneously reads *dbyangs la sogs pa’i* for *svaraniṣpannāḥ*.

^xl^ The Sanskrit manuscript’s reading *sparśādimudrāmudritāḥ* is difficult to accept, given that these four goddesses have no obvious connection to a list beginning with *sparśa*. T_D_, more plausibly, reads *pukka sī la sogs pa’i rang bzhin du rgyas btab pa*, with the reading *rim bzhin* in T_P_ and T_N_ instead of *rang bzhin*. The four goddesses indeed share their *mudrā*s with Pukkasī, Śabarī, Caṇḍālī, and Ḍombi, as taught in HeTa 2.4.16–19 and justified in 2.4.87–88. While a conjecture following exactly the Tibetan translation would be somewhat invasive, the more minimal conjecture of *pukkasyādimudrāmudritāḥ* seems reasonable. It is not implausible that *sparśā°* was accidentally copied from the *sparśa°* appearing in the following sentence.

^xli^ T_D_ erroneously renders *bhinnāñjanābhā* as *dbyer med pa’i mig sman nag po’i mdog lta bu*, as if reading *abhinnāñjanābhā*. A more common rendering by Tibetan translators for *bhinnāñjana* is *stang zil bcag pa* (see, for example, the seventh chapter of *Lalitavistara*, ed. p. 105 and D fol. 57r). On the meaning of the compound and examples from literature, see Vogel, 1967.

^xlii^ T_D_ lacks a reflex of *piṅgala* in *jvalitapiṅgalordhvakeśāḥ*: *skra ’bar ba gyen du brdzes pa*.

^xliii^ In place of *tāluke*, T_D_ reads *spyi bor*, reflecting *mūrdhni* or something equivalent. On the face of it, T_D_ strikes me as more plausible, but I have yet to find a parallel in other Hevajra or Nairātmyā *sādhana*s and thus do not feel confident in making changes to the transmitted text. In other Hevajra-related *sādhana*s, it is usually the ash smeared on the deity’s body that is said to have the nature of Vajrasattva, such as in Advayavajra’s *Hevajraviśuddhinidhi*: *vajrasattvarūpeṇa bhasmoddhūlitam* (fol. 77v1).

^xliv^ The *karmadhāraya* compound *pañcabuddhasvabhāvaśuṣkapañcamuṇḍāni* is somewhat awkward but perhaps does not require emendation.

^xlv^ cf. *Hevajraviśuddhinidhi* fol. 78r1–2: *pañcadaśasvaraviśuddhyā muṇḍamālā*.

^xlvi^ T_D_ renders *vyāghracarmāvṛtakaṭinitambāḥ* as if reading *vyāghracarmanivasanāḥ*: *stag gi pags pa’i sham thabs can*.

^xlvii^ See the following note on the reading *sthitaḥ*.

^xlviii^ The verse and a quarter labelled here 1-2a correspond to HeTa 1.6.11–12a, and these *pāda*s, along with 2b, are found in Saroruhavajra’s *Sādhanopāyikā* (p. 112) and Bhadrapāda’s *Dveṣavajrasādhana* (p. 358). Given the lack of other parallel material for 2b (another instance is *Ācāryakriyāsamuccaya* ms-g fol. 183r7–8; ms-k fol. 10r1–2 [foliation in Japanese; part of second bundle of folios]; D fol. 201r), it is likely that Advayavajra is here drawing on the *Sādhanopāyikā*.

In 1d, *smṛtaḥ* is the reading found in all palm-leaf witnesses of the HeTa available to me, and this is reflected in the Tibetan translations of the tantra (D fol. 7r) and the NaiPra: *rnam par snang mdzad brjod*. The reading is also found in the *Saṃpuṭatantra* (5.4.33d) and in the *Vajrāvalī* (p. 452), as well as in Ḍombīheruka’s *Amṛtaprabhā* as found in Bhattacharyya’s edition of the *Sādhanamālā* (p. 447). Witnesses of Saroruhavajra’s *Sādhanopāyikā*, including its Tibetan translations, and of Bhadrapāda’s *Dveṣavajrasādhana* support *sthitaḥ*. I tentatively propose that the reading *sthitaḥ* should be maintained in the NaiPra, despite its Tibetan translation, which here appears to have been influenced by ’Brog mi’s translation of the root tantra. However, the two witnesses of the *Ācāryakriyāsamuccaya* that I have consulted also support reading *smṛtaḥ*.

The word *tathā* in 2b is supported by T_D_: *lus kun rdo rje ’dzin bzhin no*. For the parallels, Gerloff’s edition of the *Sādhanopāyikā* reads *vaset*, which is reported to be found in one paper witness, while *paśyet*, unmetrical and ungrammatical, is the reading of the *Hevajrasādhanasaṅgraha* codex for both the *Sādhanopāyikā* and the *Dveṣavajrasādhana*. The canonical Tibetan translation of the *Sādhanopāyikā* reads *yan lag kun spyod rdo rje ’dzin* (Gerloff, 2020, vols. 1, 135), while a para-canonical translation reads *yan lag kun la rdor ’dzin dgod* (Gerloff, 2020, vols. 2, 152) (no Tibetan translation of the *Dveṣavajrasādhana* has been identified). The latter reading probably corresponds to what is found in the *Ācāryakriyāsamuccaya*, which reads *sarvāṅgeṣu vajradhṛk nyaset* (also grammatically anomalous). In sum, I think it is difficult to regard any of the possible readings for the final word of 2b as particularly secure.

^xlix^ The *pāda*s labelled 2c–4b correspond to HeTa 2.6.3–4d but with certain variants. To assess these individually, let us first review the verses from the tantra, alongside readings from certain of its manuscript witnesses (which are of interest here), and then review the Tibetan translation of the NaiPra.

gurvācāryeṣṭadevasya namanārthaṃ cakrikā dhṛtā |

durbhāṣasyāśravaṇāya guror vajradharasya ca || 2.6.3 ||

**3a** gruvā°] *unreadable in* P **3b** namanārthaṃ] C Nb (°rthañ) K E; namanārthā Na; navanārthaṃ P **3b** cakrikā] Σ_C_; cakṛkā C **3c** °āśravaṇāya] C Nb K E; °a sravaṇāya Na; °āśramaṇāya P **3d** guror] C Nb P E; guro Na K

śravaṇayoḥ kuṇḍalaṃ dhāryaṃ mantraṃ japtuṃ ca kaṇṭhikā |

rucakaḥ prāṇivadhaṃ tyaktuṃ mudrā bhajituṃ ca mekhalam | 2.6.4a–d

**4a** śravaṇayoḥ] C Nb K E; sravaṇayo Na P **4a** kuṇḍalaṃ dhāryaṃ] C, Nb (°lan dhāryam) P (*image unclear*) E; kuṇḍalaṃ dhārya Na K (°ryya) **4b** mantraṃ] Nb (mantrañ) P E; mantra C Na K **4b** japtuṃ] Σ_K_; japtaṃ K **4c** rucakaḥ] C Na Nb K; rucakaṃ P E **4c** prāṇivadhaṃ] Σ_E_; prāṇivandhaṃ E **4d** mudrā] Σ_E_; mudrām E **4d** bhajituṃ ca] C^pc^ N (bhajituñ ca) E; bhajas tu Na; bhañjika P; bhañjituñ ca K

For reference, the NaiPra’s verses in T_D_ run as follows:

bla ma slob dpon ’dod lha la ||

phyag ’tshal spyi bor ’khor lo ’dzin || (2cd)

bla ma rdo rje ’dzin pa la’ang ||

smod tshig mi nyan pa yi phyir ||

rna ba dag la rna cha ’dzin ||

sngags bzlas phyir ni mgul ba’i phreng || (3)

phyag rgya bsten pa ske rags te ||

srog gcod spangs pa gdu bu ste || (4ab)

In 2d (= HeTa 2.6.3b), we find *śirasi* in the Sanskrit and Tibetan of the NaiPra, but here there no evidence for it in the HeTa. The word *dhṛtā* is reflected in T_D_, as in the HeTa. A very hypermetrical reading for the *pāda*, such as *namanāya śirasi cakrikā dhṛtā*, is not implausible for the language of the tantra, but I am hesitant to adopt it for the *sādhana* unless stronger evidence becomes available.

Also in 2d (= HeTa 2.6.3b), we find *namanāya* in the NaiPra’s manuscript instead of the tantra’s *namanārthaṃ*, and similarly, instead of *mantraṃ japtuṃ* found in HeTa 2.6.4b, the NaiPra appears to read *mantrajāpāya* in 3d. The NaiPra’s readings in these cases are equivalent to those of the tantra metrically and in terms of sense.

In 3a (= HeTa 2.6.3d), it is interesting to note that, like the NaiPra’s Sanskrit manuscript, one witness of the HeTa also reads *°āśramaṇāya*, although the reading does not make very good sense and should probably be rejected.

In 3c (= HeTa 2.6.4a), it appears that Advayavajra may have replaced the tantra’s *śravaṇayoḥ* with *karṇayoḥ*, which is metrically smoother.

The *pāda*s 4a and b evidently reverse HeTa 2.6.4c and d, and this is also reflected in T_D_. The metre is rough in 4b but probably passable if *rucakaḥ* is pronounced with the right emphasis. Finally, while the tantra’s manuscripts point towards reading *mudrā* (probably to be understood as *mudrāḥ*, accusative plural), it seems equally possible that Advayavajra wrote *mudrām*, as the NaiPra’s manuscript indicates.

^l^The manuscript’s reading *nūpurakeyūradharāḥ* presents severe metrical problems: namely, it is a bad *bha-vipulā*, and the second and third syllables are *laghu*. This metrical configuation is highly implausible for the writing of Advayavajra and even, I would suggest, in most scripture. The *pāda*’s content also seems out of place among these verses, which are no longer focused on describing the goddesses. However, the Tibetan translation supports the manuscript’s reading and indicates a *pāda* of *anuṣṭubh*, although the metrical details are of course not evident: *rkang rgyan dang ni dpung rgyan ’dzin ||*

Although it goes against the Tibetan, reading *keyūranūpuradharāḥ* yields an acceptable *na-vipulā*. But without parallel attestation, doubts remain concerning the passage. It is possible that the *pāda* has its origins in a scripture that is currently unavailable to us.

^li^ 4d corresponds exactly to HeTa 2.9.11b. Note that Snellgrove’s edition of the tantra reads *maitricittataḥ*, with no variants reported. The more expected *maitrīcittataḥ* would be unmetrical, but all palm-leaf manuscripts of the tantra that are available to me support *maitracittataḥ*, as do citations of the *pāda* in Ratnākaraśānti’s *Bhramaharasādhana* (p. 166) and the *Sādhanopāyikā* (p. 111).

^lii^ T_D_ renders this sentence in verse.

^liii^ The reading *mahārāgatā* is not impossible if understood as ‘great redness’, but it seems likely that Advayavajra would use a different word here, given that he just used *mahārāga* in the sense of ‘great passion’. The conjecture *āraktatā* can also be considered. cf. *Hevajraviśuddhinidhi*: *kṛpayā kāyavākcittavajrarūpāṇāṃ netrāṇām āraktatā* (fol. 77r6).

^liv^ cf. HeTa 1.8.20ab: *tathā mānādiṣaḍdoṣān kartituṃ kartikā sthitā* |

^lv^ For *caturmārarudhirapūrṇaṃ*, T_D_ suggests reading *°caturmārādirudhirapūrṇaṃ*: *bdud bzhi la sogs pa’i khrag gis bkang ba |*

^lvi^ cf. *Hevajraviśuddhinidhi*: *bhāvābhāvavikalpanaśarīraśiraḥviśuddhyā padmabhājanam* (fol. 78r1). If the text of the NaiPra is constituted correctly here (it may not be), perhaps we are to understand *sakalavikalpaśarīri kapālaṃ* as meaning ‘the head whose body [was] all conceptual thought’. cf. HeTa 1.8.20cd: *bhāvābhāvavikalpasya śirasā padmabhājanam* | These *pāda*s can be understood as, ‘The skull cup has [as its pure nature (*viśuddhi*)] the head of conceptualisation regarding existence and non-existence.’

^lvii^ The *tribhaṅga* is also mentioned in Advayavajra’s *Hevajraviśuddhinidhi*: *nirmāṇadharmasambhogakāyatrayaviśuddhyā tribhaṅgaṃ* (fol. 77v6). The word is rare in Buddhist texts but can also be found in Abhayākaragupta’s *Vajrāvalī* (p. 184), describing *patāka*s in the compound *anilāndolanatribhaṅgalolābhinayāḥ*. For the NaiPra, T_D_ renders the word as *gnyer ma gsum mo*, which misleadingly suggests the Sanskrit *trivalī*. ’Gos gZhon nu dpal’s Tibetan translation of the *Hevajraviśuddhinidhi*’s renders it *sum khyog* (Dg fol. 186f3) and the translation of the *Vajrāvalī* as *sum khugs* (p. 185). The word was not rendered in the anonymous translation of the *Hevajraviśuddhinidhi* (Da fol. 172v3).

^lviii^ T_D_ renders *svābhāvikakāyaviśuddhyā* as *ngo bo nyid gcig pu’i sku’i rnam par dag pa*. The *gcig pu* is difficult to account for, but perhaps is the result of reading (erroneously) *svabhāvaikakāya°*.

^lix^ That the deity is *ālambanaikacaraṇa*, or ‘one who has a single foot as a support’, refers to her *ardhaparyaṅka* stance; cf. *Hevajraviśuddhinidhi*: *sakalatraidhātukanirālambaviśuddhyā ardhaparyaṅkatā* (fol. 77v6–7).

^lx^ The manuscript’s reading *nairātmyāsamayaḥ* can be emended to *nairātmyāsamaḥ* not simply because of support from T_D_ (*bdag med ma dang mnyam par*) but also on the grounds of sense. The practitioner is already *nairātmyāsamaya* insofar as he has been visualising himself as the goddess; however, only after dissolving the *jñānasattva* into that visualisation does he become *nairātmyāsama*. An alternative possible emendation is *nairātmyāmayaḥ*. The same phase of the *sādhana* is described in Advayavajra’s *Hevajraviśuddhinidhi* as follows: *samayacakrajñānacakrayor jale jalam ivaikalolībhāvaṃ cintayet* (fol. 72r4–5).

^lxi^ cf. HeTa 1.8.22c–24b: *prathamaṃ bhāvayet kṛṣṇāṃ dvitīye raktām vibhāvayet* || 22 || *tṛtīye bhāvayet pītāṃ caturthe haritakāṃ tathā | pañcame nīlavarṇāṃ ca ṣaṣṭhame śukladehikām* || 23 || *ṣaḍaṅgaṃ bhāvayed yogī viramāntaṃ punas tathā |* 24ab

^lxii^ cf. HeTa 1.10.11–12ab: *prathamaṃ meghavad bhāti siddhe tu māyāvad bhavet* | *sahasā svapnavad bhāti svapijāgradabhedavat* || 11 || *abhedalakṣaṇāsiddhau mudrāyogīti sidhyati* | 12ab

T_D_ renders *svapnajāgraddaśayor abhedaprāpto mahāmudrāyogī sidhyati* somewhat differently, including a relative pronoun and reading *mthong ba* where one might expect *thob pa*: *rmi lam dang sad pa ’dra ba’i dus su gnyis su med par gang gis mthong bas phyag rgya chen po thob par ’gyur ro ||*

^lxiii^ Mathes (2014, p. 374) (also Mathes, 2021, p. 132), while discussing the possibility of Advayavajra advocating a so-called *sūtra-mahāmudrā*, interprets this passage as not simply presenting different 'options' or types of Nairātmyā-related practices that are generally available, but also allowing for optionality in the sequentiality of these practices and, by extension, the sequentiality of empowerments. Mathes writes: ‘… it is not completely out of the question that an empowerment in Maitrīpa's system could start directly with the *prajñājñāna*-empowerment. In his *Nairātmyāprakāśa*, Maitrīpa thus explains the ordinary creation stage as an optional practice, and not as a necessary requirement for the subsequent stages.’ Although the NaiPra evidently allows for some optionality with respect to the five stages of practice it outlines, this does not necessarily entail that these practices lack different requirements (such as different *abhiṣeka*s) or that a particular stage is not still a required preliminary for another (e.g., the *utpattikrama* may still be a requirement for the *gambhīrotpattikrama* while remaining optional for more advanced practitioners).

^lxiv^ T_D_ renders *virama* as *bral ba*, which may be an acceptable translation but diverges from the more common renderings of this technical term as either *khyad dga’* or *dga’ bral*.

^lxv^ For *°pañcadaśayoginīrūpaṃ paśyet*, T_D_ may reflect the reading *°pañcadaśayoginīmaṇḍalacakraṃ paśyet*: *rnal ’byor ma bco lnga’i dkyil ’khor gyi ’khor lor blta bar bya’o ||*

^lxvi^ This passage (beginning *nairātmyāhaṃkāram udvahan*) has been translated in two publications by Mathes (2014, pp. 373–374; 2021, pp. 132–133). In the former a draft edition of the passage by isaacson is included in a footnote; and the latter publication also includes a translation of the sentence below that begins *anābhogayuganaddhādvayavāhi*.

^lxvii^ The reading *anavalokayann* is perhaps not entirely secure, with *avalokayann* also a possibility. Neither is reflected in T_D_. It is possible that the *utpannakrama* here invoves manifesting the deity’s circle without so much as visualising a seed syllable. However, there are also descriptions of the *utpattikrama* which involve intense focus on a subtle element such as a seed syllable, which could cause the spontaneous manifestation of the *maṇḍala*. cf. Ratnākaraśānti’s MuĀ ad HeTa 1.1.11: *api ca tasya mahājñānasya bhāvanārthaṃ mudrāpi sarvabuddhair adhiṣṭhitā, tadyathā sūkṣmaḥ samagro hūṃkāraḥ svaracandramātrārahito vā bindumātraṃ vā sarṣapasūkṣmaṃ paramasūkṣmaṃ vā catuścakrabhedena vā catvāry akṣarāṇi saparicchadāni. mudrāpakṣe utpattikramaḥ prāpnoti notpannakrama iti cet—naitad asti. utpattikramaśabdo hy atra pāribhāṣiko na laukikaḥ. sa ca mantracihnādipariṇāmajaṃ devatādeham āha na mantramātram api* (ed-i p. 483).

^lxviii^ In place of *vajraśarīre khalu jñānādhiṣṭhite*, T_D_ suggests reading *pañcajñānādhiṣṭhitāḥ* as an adjective describing *nāḍyaḥ*: *rdo rje’i lus kyang ye shes lngas byin gyis brlabs pa’i rtsa sum cu rtsa gnyis te |* Ṅ’s reading is slightly more convincing: that the body is presided over by *jñāna* is frequently and famously expressed in the HeTa—for example, 1.1.12: *dehasthaṃ ca mahājñānam*. I also do not immediately see why the five forms of *jñāna* are relevant here.

^lxix^ The passage beginning *tathā hi lalanārasane* strongly resembles a passage in Ratnākaraśānti’s MuĀ ad HeTa 1.1.16: *lalanārasane eva kaṇṭhād ārabhya yāvan nābhiḥ. atrāntare vāmetarapārśvanāḍyau candrasūryākhye. nābher adhas te eva yonināḍyau lalanārasanākhye eva* (*lalanārasanākhye*] ms-a ed-t; *lala...* ms-b [lost to damage]) (ms-a fol. 17r; ms-c 12v; ed-t p. 19).

^lxx^ For *snāyvasthimālāvahe*, T_D_ reads *rgyus pa dang | rus pa dang | dri ’bab ba dag*. Perhaps the translator erroneously read or understood *snāyvasthimalāvahe*.

^lxxi^ For *vṛkkahṛdayavahe*, T_D_ reads *glo ba dang snying ’bab pa ste*. One expects *mkhal ma* for *vṛkka*, which here refers to the kidneys.

^lxxii^ For *phupphusāntramālāvahe*, T_D_ reads *mchin pa dang rgyu ma dang dri ’bab pa*. The rendering *glo ba* would be expected for *phupphusa*, and the translation again reflecs *mala* instead of *mālā*.

^lxxiii^ For this *nāḍī* sources give the names *hṛṣṭavadanā* and *kṛṣṇavadanā* (the *akṣara*s for *hṛ* and *kṛ* having similar forms in North Indian scripts). Here T_D_ reads *mdog nag ma*, which may be a transmissional error for *mdong nag ma* and therefore a reflection of the latter.

^lxxiv^ This sentence closely resembles the phrasing of the MuĀ ad HeTa 1.1.17: *hṛṣṭeti hṛṣṭavadanā. sā liṅge sīmantamadhyagā* (*sīmantamadhyagā*] em.; *sīmāntamadhyagā* ms-c ed-t; *sīma<ā>ntamadhyagāḥ* ms-a) (ms-a fol. 18r; ms-c fol. 13v; ed-t p. 20). Based on this we can feel confident emending the manuscript’s reading of *hṛlliṅge* to *liṅge*.

^lxxv^ For *svarūpiṇīsāmānye*, T_D_ reads *phra gzugs ma dang spyi ma dag*, as if reflecting *sūkṣmarūpiṇī*.

^lxxvi^ Where the Sanskrit manuscript reads *medaḥkheṭavahe*, we expect the second member of this compound to be a word meaning ‘tears’. T_D_’s reading *mchin pa* is likely a scribal error for *mchi ma*. The conjecture *medośruvahe* is plausible, but *medaḥkhedāśruvahe* is a more likely cause of error. Kamalanātha, in his *Ratnāvalī* (fol. 3r7), uses the word *śokāśru* in this context, for which *khedāśru* can be regarded as equivalent.

^lxxvii^ Here where the Sanskrit manuscript reads *lasiṃhāṇa°*, I conjecture *bālasiṃhāṇa°* on the basis of *Vasantatilaka* 6.39 (also *Saṃpuṭatantra* 6.2.32), as well as on the basis of MuĀ ad HeTa 1.1.17: *sumanā jānudvaye bālasiṃhāṇavahā* (*bālasiṃhāṇavahā*] ms-c ed-t [°*siṅghāṇa*°]; *bālasiṅghānakāvahā* ms-a) (ms-a 18v; ms-c fol. 13v; ed-t p. 21). Perhaps *bāla* is to be understood in the sense of a child, and something akin to this understanding is reflected in the *Vasantatilaka*’s Tibetan translation, which renders *bālasiṃhāṇa* as *byi pa’i sna ba* (D fol. 302r). It also seems possible to understand *bāla* (or *vāla*) in the sense of hair. The MuĀ’s Tibetan translation, however, renders the compound in question as *kha chu dang snabs* (D fol. 232r), which suggests reading *lālāsiṃhāṇa*. This reading is attractive and a common combination in Sanskrit, but I have not seen support for it in Sanskrit witnesses of the passages in question. The NaiPra’s Tibetan translation reads *stobs dang snabs*, and the Tibetan translation of the *Saṃpuṭatantra* reads *sha dang snabs*, both of which could suggest reading *balasiṃhāṇa*, but again I have yet to see a Sanskrit witness support this. The matter remains somewhat in doubt.

^lxxviii^ This sentence is found in Ratnākaraśānti’s MuĀ ad HeTa 1.1.14: *tatra yā nāḍī yaṃ prasūte puṣṇāti gacchati vā sā tadvahā yathāyogam* (*tatra yā nāḍī yaṃ*] ms-a ed; *tatra nāḍī | nāḍī yaṃ* ms-c) (ms-a fol. 17v; ms-c fol. 13r; ed-t p. 20). T_D_ is problematic here: *’di yang rtsa nas rab tu ’dzag pas | rgyas par byed pa dang | ’gro bar byed pa dang | de nas cung zad ’bab pa’o ||* I am not certain what the translator intended by this formulation, but there appears to have been some confusion on his part. He does appear to have read *prasṛte*, which is also the reading of the NaiPra’s Sanskrit manuscript. I believe the MuĀ’s reading is superior here, as it provides a clearer explanation of the channels’ relationship to the substances, and because it would otherwise be difficult to differentiate *prasṛte* and *gacchati* in meaning.

^lxxix^ A few elements are missing from this sentence in T_D_—namely, *kiṃ ca*, *etāḥ*, and *°tribhava°*: *sku dang gsung dang thugs dang | chos dang longs spyod rdzogs pa dang | sprul pa’i ngo bo nyid ni ’khor lo bzhir lus la rnam par gnas pa’o ||*

^lxxx^ For *viśvavarṇacatuḥṣaṣṭidalaṃ*, T_D_ suggests including a word such as *padma* or *kamala*: *kha dog sna tshogs pa padma ’dab ma drug cu rtsa bzhi pa*. In the following sentences we have similar compounds: *śuklāṣṭadalakamalaṃ*, *raktaṣoḍaśadalaṃ*, and *śukladvātriṃśaddalaṃ*. For the latter two compounds, T_D_ shows no reflex of a word meaning ‘lotus’. With or without such a word, all of these compounds are cogent, so an emendation to the first based on T_D_ is not strongly compelling. For the first of these four compounds, Ṅ reads *viśvavarṇaṃ catuḥṣaṣṭidalaṃ*, but the subsequent compounds suggest that the colour should be part of the compound.

^lxxxi^ T_D_ lacks a reflex of *śukla* in *śuklāṣṭadalakamalaṃ*: *padma ’dab ma brgyad pa*

^lxxxii^ Where the present edition reads *anābhogayuganaddhādvayavāhi bodhicittasākṣātkaraṇaṃ svābhāvikaḥ kramaḥ* (*anābhogayuganaddhavāhi* in the manuscript), there are a few points to consider in reference to T_D_: *’bad pa med par zung du ’jug pa gnyis med par ’byung ba’i byang chub kyi sems mngon du byed pa’i rgyu’o || ngo bo nyid kyi rim pa’o ||* First, syntactically T_D_ understands two sentences where I understand only one. T_D_ has perhaps confused *karaṇa* with *kāraṇa*. It also reflects the word *advaya*, not found in the Sanskrit manuscript, within the compound ending *vāhin*, and it connects this compound with the following word, thus qualifying *bodhicitta*. These last two points are valid possibilities, and of them I wish to accept the former. We find a few parallels in Advayavajra’s corpus for the compound *yuganaddhādvayavāhin*: e.g., in the *Amanasikārādhāra* (p. 497), the *Sekatātparyasaṅgraha* (p. 413), and the *Pañcatathāgatamudrāvivaraṇa* (p. 377). In the *Sekatātparyasaṅgraha*, however, we also find *yuganaddhavāhin*, as part of the compound *asaṃkṛtābhedayuganaddhavāhibodhasamaya*. In general, however, it appears that Advayavajra preferred the formulation that includes *advaya*.

Whether *anābhogayuganaddhādvayavāhin* should qualify *bodhicitta* or *sākṣātkaraṇa* is slightly more difficult to determine, but perhaps ultimately there is no great difference. *Bodhicitta*, the innate nature of mind, is *anābhogayuganaddhādvayavāhin* in that it supports (*vāhin* in the sense of ‘bearing’) the non-dual state of the effortless coalescence of bliss/compassion and emptiness; manifesting *bodhicitta* is *anābhogayuganaddhādvayavāhin* in that it produces/leads to (*vāhin* in the sense of *pra-√sū* etc.) the non-dual state that is effortless coalescence. Note that the above-quoted *Sekatātparyasaṅgraha* lends support to the latter interpretation.

^lxxxiii^ Where the Sanskrit manuscript reads *svāhāntāḥ*, T_D_ reads *hūṃ phaṭ mthar gnas pa* and indeed ends the mantra with *hūṁ phaṭ svāhā*.

^lxxxiv^ The manuscript’s *svābhāṅganāṃ* may point to reading *svābhāṅganā°*, while T_D_ suggests *svāṅga°* or *svāṅgasya* with *rang gi yan lag gi*. On the level of sense, the latter may at first appear more appealing, since here it is the practitioner, identifying as Nairātmyā, who is transforming the perception of his own body. However, it is also not unreasonable to refer to Nairātmyā as the practitioner’s *svābhāṅganā*, a very common term in Buddhist tantric literature for one’s personal consort. And given the prominence of this term, it is also reasonable to believe that this reading is original.

^lxxxv^ Where the Sanskrit manuscript reads *cakranayā śāstrasāraṃ raṃ*, I hesitantly propose the conjecture *mantrayānaśāstrasāraṃ* with some inspiration from T_D_: *gsang sngags tshul gyi bstan bcos snying ||* We might expect *mantranaya* for *gsang sngags thsul*, but that would be metrically bad. A reading such as *mantrayāne śāstrasāraṃ* is also worth considering. Other possibilities include reading *mantrāmnaye, mantrāmnaya°*, or any of the preceding with *vajra* in place of *mantra*. Assuming *śāstrasāraṃ raṃ* is a mistake for *śāstrasāraṃ*, the verse has a *ra-vipulā* and requires a caesura after the *pāda*’s fourth syllable. The *Dhīḥ* edition proposes reading *cakranayaśāstrasāraṃ*, which is unmetrical and does not yield good sense—*cakranaya* is not, as far as I am aware, a synonym for the *mantranaya*.

^lxxxvi^ For the second *pāda* of this verse, T_D_ reads *bla ma dam pa’i ’bad pa las shes pa*. If this transmitted reading is what was written by the translator, it reflects understanding *guroḥ* as a genitive form connected to *yatnena*, yielding a bizarre meaning, ‘having understood the essence of the *śāstra*s of the Way of Mantra by means of [my] teacher’s effort’.

^lxxxvii^ Where the Sanskrit manuscript reads *rūkṣitamastako*, I conjecture *rūkṣitamastakatā* for the verse to be metrical. It is evidently in Drutavilambita. The *Dhīḥ* edition accepts the unmetrical reading.

T_D_ translates: *dkyil ’khor ’khor lo zab mo yi ||rnam par nges par ’gyur ba ni || ji ltar ’dir ni lus can rnams || ri khrod mgon gyi zhabs kyi chu skyes kyi || rdul rnams spyi bos ma blangs pa || de dag nges par ji ltar ’gyur ||* We see no reflex of *yadi* in the second half of the verse, and the final *pāda* does not seem to make sense or be based on anything we have in Sanskrit.

^lxxxviii^ This verse, in Puṣpitāgrā metre, is transcribed in Isaacson (2009, 128), where the conjectural addition of *bhavet* is proposed. The diagnostic conjecture I offer here for the first *pāda* is not reflected in T_D_; its reading suggests *anenābhisamayena* or another permutation, which is hard to make work within the given metrical constraints and is not clearly related to the letters found in the Sanskrit manuscript. The entire verse in Tibetan runs as follows: *mngon par rtogs pa ’di yis thob pa yis || dge ba ’di yis ’jig rten mtha’ dag ni || srid pa gsum gyi sdug bsngal yid mi bde spangs te || rdo rje ’dzin pa’i go ’phang rab gnas shog ||*

## Footnotes

1 I would like to thank Harunaga Isaacson for carefully reading the *Nairātmyāprakāśa* with me and for later offering invaluable detailed feedback on all aspects of this article. I would also like to express my gratitude to the other half a dozen or so colleagues who attended our reading sessions, as well as to Orna Almogi for reading a draft of the introduction. This research was funded by the European Research Council (ERC) Synergy project Intellexus, grant no. 101118558.

2 This date range is argued for by Isaacson and Sferra (2014, p. 71). Tatz (1987, pp. 697–98) earlier proposed the precise dates of 1007–1085, which Roberts (2011, p. 618 n. 20) suggests adjusting to 986–1063. But as Isaacson and Sferra point out, such precision is based on much later, and at present impossible to verify, Tibetan sources.

3 My thanks to Harunaga Isaacson for pointing this out to me.

4 For this and other references, see the Nairātmyā entry in the Indo-Tibetan Lexical Resource, https://www.itlr.net/hwid:105571, accessed November 1, 2024.

5 One text included in the Peking bsTan ’gyur, *bDag med ma’i byin rlabs* (Ōta. 4706), is not an Indic text but is by Zhe ldang rdo rje, a student of Mar pa Chos kyi blo gros also known as rNgog mDo sde.

6 Advayavajra likely also had a notable impact on Buddhism as practised in the Kathmandu Valley, at least judging by how his works were incorporated into Newar-compiled scriptures such as the *Yogāmbaratantra* and the *Nairātmyātantra*. See the note below on the *maṅgalācaraṇa* as well as (Szántó 2016).

7 This number is based solely on the sDe dge bsTan ’gyur. It includes works ascribed simply to an ‘Avadhūta’ (potentially referring to Advayavajra or any figure with this appellation), as well as six works for which he or a namesake appears as a translator’s assistant *paṇḍita*. Works with multiple Tibetan translations are counted only once. While many of these works have been demonstrated not to be by the author in question, the authorship of many remains uncertain and merits further investigation.

8 The relevant portion of the *Hevajrākhya* reads: *tataḥ paṭhej jinamantrakam—*oṁ śūnyatājñānavajrasvabhāvātmako ’ham. *tasmin samaye svapara*(*ṃ*) *śaradamalanabhasannibhaṃ paśyet* (fol. 9r).

9 In his **Caturmudropadeśa*, Advayavajra refers to this as the **bāhyotpattikrama* (*phyi’i bskyed pa’i rim pa*) (p. 387 ff.), distinguishing it from the *gambhīrotpattikrama*.

10 Schaeffer (2005, 65) has written on the provenance of this text’s Tibetan translation, which may have been transmitted through Vajrapāṇi.

11 Divākaracandra, Vajrapāṇi, Rāmapāla are counted amongst Advayavajra’s four ‘great’ disciples as per several Tibetan sources, the remaining disciple being Natekara/*Naṭegana (a.k.a. *Sahajavajra [Lhan cig skyes pa’i rdo rje]) (Isaacson and Sferra 2014, 82). ’Gos gZhon nu dpal reports that *Kāropāda (a.k.a Vītakarman and Karopa) is also said to have been a student of Advayavajra (Sanderson 2009, 223 n. 586).

12 For instance, in describing the *maṇḍala*’s protective features, Ratnākaraśānti writes: *viśvavajrakiraṇaiḥ pralayānaladuḥsahaiḥ sarvataḥ spharitvā ghanībhūya racitaṃ tiryak caturasraṃ jvaladvajraprākāram upariṣṭād vajrapañjaram adhastād vajramayīṃ bhūmim ārasātalaviracitāṃ paśyet | tato raviviśvavajrābhyāṃ raśmībhūya diśi diśi spharitvā ghanībhūya bahirdūre sīmābandhaḥ karaṇīya iti ślokārthaḥ |* (*Bhramaharasādhana* p. 159–160). And Advayavajra’s *Hevajraviśuddhinidhi* reads: *tata* (*sic* for *tato*) *viśvavajrakiraṇaiḥ pralayānaladuḥsahaiḥ sarvataḥ spharitvā ghanībhūya caturasra*[*ṃ*] *yāvadicchāvistaraṃ jvaladvajraprākāraṃ upariṣṭād vajrapañjaraṃ || adhastād vajramayī*[*ṃ*] *bhūmiṃ ārasātalaviracitāṃ paśyet || tato raviviśvavajrābhyāṃ raśmībhūya diśi diśi sphritvā tat sarvaṃ dṛḍhīkuryāt ||* (fol. 66v4–6)

13 This team also worked on six other texts by Advayavajra: the *Kudṛṣṭinirghātana* (Tōh. 2229), its *pañjikā* (Tōh. 2231), the *Sahajaṣaṭka* (Tōh. 2232), the *Tattvadaśaka* (Tōh. 2236), the *Sekatātparyasaṅgraha* (Tōh. 2243), and the *Premapañcaka* (Tōh. 2246). They also translated a *homaprakriyā* by Kokadatta (a.k.a. Koṅkadatta) connected to Vajravārāhī (Tōh. 1576) and Vajrapāṇi’s own compilation of *vajrapada*s (Tōh. 2255).

14 This is not the case for the *Dhīḥ* edition, which, for example, prints the impossible *nairātmyāhaṃkāram udvahanamantraṃ japet*, whereas I silently allow the reading to be *udvahan mantraṃ*.

15 spaṣṭaṃ sabodhaṃ] *conj.*; spaṣṭaṃm abodha((ṃ)) Ṅ (*the possible* anusvāra *is unusually shaped and placed far to the* akṣara*’s left*)

16 śmaśānādau] *em.*; śmaśānādi Ṅ

17 sukhāsanopaviṣṭo] *em.*; sukhāsanopaviṣṭaṣṭo Ṅ

18 niḥśaṅkaḥ] *em.*; niḥśaṅka Ṅ

19 sattvārthodyatamatir] *em.*; satvā((r))thodyamati Ṅ

20 tatas tadīyai] *conj.*; tatas thadīyai Ṅ; de nas de’i gsal ba’i T_D_ (tatas taddīptai — *possible conjecture*)

21 alimalinetara°] *conj.* (isaacson); alamalinetara° Ṅ; shin tu gnag pa T_D_ (atimalinetara° — *possible conjecture*)

22 °liṅgitakandharam] *em.*; °liṃgitaṃkandharam Ṅ

23 ambaratala°] *em.*; ambaratarala° Ṅ

24 bāhyaguhyatattvapūjābhir] *em.* (T_D_: phyi dang | gsang ba dang | de kho na nyid rnams kyis mchod pa); bāhyapūjāguhyatatvapūjābhir Ṅ

25 tādātmyāvabodhanāya … °cittotpādātmabhāva°] *diag. conj.* (T_D_ [*starting from* atra ca *and ending* ca kṛtvā]: ’di la yang thabs dang shes rab dag gis de’i bdag nyid rtogs par bya ba’i phyir pi tsu badzra’i mchod pa’o || de nas phyag ’tshal ba dang | sdig pa bshags pa dang | sdig pa slan chad sdom pa dang | bsod nams la rjes su yi rang ba dang | bsod nams yongs su bsngo ba dang | gsum la skyabs su ’gro ba dang | byang chub tu sems bskyed pa dang | bdag nyid kyi dngos po dbul ba dang | gsol ba gdab pa byas te |); tādātmabhāva° Ṅ

26 °ādhyeṣaṇāś] *em.*; °ādhyeṣyaṇāś Ṅ

27 °saṅgrāhakātmakaṃ] *em.*; °saṅgrahākātmakaṃ Ṅ

28 sphuradbhir aṃśusaṃhater vajrair] *conj.*; sphuradbhir aṇusaṃhater vvajraiḥ Ṅ; ’od zer gyi rdul phra rab kyi tshogs ’phros pa des T_D_

29 vajraprākāraṃ] *em.*; vajraprākārai Ṅ

30 bhūmiṃ] *em.*; bhūmi Ṅ

31 vicintayet] *em.*; viñcintayet Ṅ

32 ūrdhvaviśālāṃ] *conj.* (T_D_: steng yangs pa); ūrddhvāṃ Ṅ

33 °āṣṭadalaṃ] *em.*; °āṣṭadala Ṅ

34 viśālaṃ] *em.*; viśāla Ṅ; *not reflected in* T_D_

35 viśvavajramadhye] Ṅ^pc^; vijaśvavajramadhye Ṅ^ac^

36 °jalāvanimaṇḍalāni] *em.*; jalāvani Ṅ (T_D_: chu dang | sa rnams ni)

37 jñānamātram] *em.*; jñānamātraṃm Ṅ

38 harītakīvṛkṣe] *em.*; harītakīvṛkṣa Ṅ

39 cūtavṛkṣe] *em.*; cūtavṛkṣa Ṅ

40 āgneyyāṃ] *em.*; agneyāṃ Ṅ

41 karañjavṛkṣe] *em.*; karañja Ṅ

42 aiśānyāṃ] *em.*; aiśānyā Ṅ

43 cāṣṭau prasiddhavarṇā … yakṣavarṇāni] *diag. conj.*; cāṣṭau yakṣavarṇṇāni Ṅ; de ltar phyogs skyong brgyad kyi kha dog ni grags par zad do || mchod rten brgyad kyi kha dog kyang rtogs par bya’o || T_D_ (*starting from* evaṃ dikpālāś *and up to* boddhavyāni)

44 boddhavyāni] *em.*; bodhavyāni Ṅ

45 °yoginīr] *em.*; °yoginī Ṅ

46 dhyeyā] *em.*; dhyeyāḥ Ṅ

47 a-kārasvarasambhavā] *em.*; akārasvarasahyā Ṅ

48 ā-i-ī] *em.*; a ā i Ṅ

49 °rāgerṣyāsvabhāvā] *em.*; °rāgairṣyāsvabhāva Ṅ

50 °saṃskāra°] *em.*; °saṃskārā° Ṅ

51 °ḍākinīr] *em.*; °ḍākinī Ṅ

52 ū] *em.*; ṛ ū Ṅ

53 °svaraniṣpannā] *em.*; °svarāni<ā>ṣpannā Ṅ

54 °sambhavāmitābha°] *em.*; °sambha((ḥ))vāmitābhā° Ṅ

55 pukkasyādimudrāmudritā] *conj.*; sparśādimudrāmudritā Ṅ; pukka sī la sogs pa’i rang bzhin du rgyas btab pa T_D_

56 rūpaśabdagandharasa°] *em.* (T_D_ gzugs dang | sgra dang | dri dang | ro rnams); rūpaśabdagandha° Ṅ

57 tadanantaram] *em.*; tadanantara Ṅ

58 moharāgamudrite] *em.* (T_D_: gti mug dang ’dod chags kyis rgyas btab pa); moharāge mudrite Ṅ

59 °khecaryau] *em.*; °khecaryyā Ṅ

60 svakuleśā°] Ṅ^pc^; sva((kra))leśā° Ṅ^ac^

61 boddhavyā] *em.*; bodhavyā Ṅ

62 vajrasattvasvabhāva°] *em.*; vajrasatvasvabhāvā Ṅ

63 pañcadaśamātṛkāsvabhāvaśuṣka°] *em.* (T_D_: ma mo bco lnga’i bdag nyid kyi mgo bo skam po); pañcadaśamātṛkāsvabhāvā śuṣka° Ṅ

64 °carmāvṛta°] *em.*; °carmmāvṛtā Ṅ

65 °āmitābhaḥ] *em.*; °āmitābha Ṅ

66 °devasya] *em.*; °devatāsya Ṅ

67 °āśravaṇāya] *em.* (T_D_: mi nyan pa); °āśramaṇāya Ṅ

68 dhāryaṃ] *em.*; dhāryya Ṅ

69 keyūranūpuradharāḥ] *conj.*; nūpurakeyūradharāḥ Ṅ

70 tatsvabhāvānāṃ] *conj.*; svabhāvānāṃ Ṅ; rang bzhin gyis T_D_ (svabhāvena - *also a plausible conjecture*)

71 mahāraktatā] *conj.*; mahārāgatā Ṅ

72 trikhaṇḍaṃ] Ṅ^pc^; trimukhaṇḍaṃ Ṅ^ac^

73 sakalavikalpaśarīri] *em.* (T_D_: rnam par rtog pa mtha’ dag gis lus); kamalavikalpaśarīri Ṅ

74 tribhaṅgam] *em.*; tribhaṅgāṃ Ṅ

75 °ālambanaikacaraṇatā] *em.*; °ālambane | kacaraṇatā Ṅ; zhabs ma sbyangs pa T_D_ (*read instead* ma brkyangs pa?)

76 °vinirgataiḥ] *em.*; °vinirgati Ṅ

77 kṛtvā] *em.* (T_D_: byas nas); bhūtvā Ṅ

78 nairātmyāsamo] *em.* (T_D_: bdag med ma dang mnyam par); nairātmyāsamayo Ṅ

79 °pītaharitanīla°] *em.* (T_D_: ser po dang | ljang gu dang | sngon po dang); °pītaharitapītanīla° Ṅ

80 meghasaṃchanna°] *em.*; meghasaṃchannaṃ Ṅ

81 paramaviramamadhyagaṃ] *em.*; paramaviramadhyaga° Ṅ

82 yoginīrūpaṃ] *em.*; yoginīrūpa Ṅ

83 tasyāpi] *em.* (T_D_: de yang); tasya hi Ṅ

84 anavalokayann] *em.*; anavalokayana Ṅ; *no reflex in* T_D_; avalokayann *possible conj.*

85 dvātriṃśan] Ṅ^pc^; dvātriṃśatan Ṅ^ac^

86 kaṇṭhād] *em.* (T_D_: mgrin pa nas); karṇṇād Ṅ

87 °nābhi°] *em.*; °nābhiṃ Ṅ

88 bodhicittavahā] *em.*; bodhicittāvahā Ṅ

89 °roma°] *orthographic change*; °loma° Ṅ

90 tvaṅmalavahā] *em.*; tvajmalavaho Ṅ

91 vāmakarṇabhrūmadhyasthe] *em.*; vākarṇṇabhrūmadhyesthe Ṅ

92 °bāhumūlasthe] *em.*; °bāhumūlesthe Ṅ

93 nābhināsāgrasthe] Ṅ^pc^; nābhinā..kasāgrasthe Ṅ^ac^

94 phupphusāntramālāvahe] *em.*; phupphuṣāntranālāvahe Ṅ

95 liṅge] *em.*; hṛlliṅge Ṅ; *no reflex in* T_D_

96 meḍhragudayoḥ] *em.*; na | me<mayā>ḍhragudayoḥ Ṅ

97 pādāṅguṣṭha°] *em.*; pādāṅguṣṭhali° Ṅ

98 medaḥkhedāśruvahe] *conj.*; medaḥkheṭavahe Ṅ; tshil dang mchin pa ’bab pa T_D_

99 °sumane] *em.*; °samāne Ṅ

100 bālasiṃhāṇavahā] *em.*; lasiṃhāṇavahā Ṅ; stobs dang snabs ’bab pa T_D_

101 prathamaṃ] *em.*; prathamā Ṅ

102 śeṣāḥ khecarī] *diag. conj.* (T_D_: lhag ma rnams ni mkha’ spyod ma’o ||); *deest* in Ṅ

103 prasūte] *em.*; prasṛte Ṅ

104 caitāḥ] *em.*; cetāḥ Ṅ

105 °svabhāvāś] Ṅ^pc^ (°svabhāvāḥ |); °svabhāvāḥ | ś Ṅ^ac^

106 viśvavarṇa°] *em.*; viśvavarṇṇaṃ Ṅ

107 kṛṣṇa-a-kārabījaṃ] *em.*; kṛṣṇāṃkārabījaṃ Ṅ

108 vyavasthitam] *em.*; vyavasthita Ṅ

109 raktaṣoḍaśadalaṃ] *em.*; raktaṣoḍaśadala Ṅ

110 boddhavyā] *em.*; bodhavyā Ṅ

111 anābhogayuganaddhādvayavāhi] *conj.* (T_D_: ’bad pa med par zung du ’jug pa gnyis med par ’byung ba’i); anābhogayuganaddhavāhi Ṅ

112 mantraṃ] *em.*; mantra Ṅ

113 raktanairātmyāhaṃkāram … tadanantaraṃ maṇḍalacakrasā°] *The text beginning* raktanairātmyā° *and ending* maṇḍalacakrasā° *is written in a second hand. Therein, the text beginning* japet *is written as a marginal addition.*

114 purakṣobhamantraṃ] *diag. conj.* (T_D_: grong khyer dkrug pa’i sngags); mantram imaṃ Ṅ

115 °sākṣātkāradaśāyāṃ] *em.* (T_D_: mngon du byas pa’i dus su); sākṣāddaśāyāṃ Ṅ

116 svābhāṅganā°] *conj.* (isaacson); svābhāṅganāṃ Ṅ; rang gi yan lag gi T_D_ (svāṅga° - *possible conj.*)

117 mantrayāna°] *diag. conj.*; cakranayā Ṅ; gsang sngags tshul gyi T_D_

118 °sāraṃ] *em.*; °sāraṃ raṃ Ṅ

119 rūkṣitamastakatā] *conj.*; rūkṣitamastako Ṅ

120 abhisamayasuvistṛtau] *diag. conj.*; abhisamayavistarite Ṅ; abhisamayasuvistare *possible diag. conj.*

121 anena bhavet] *conj.* (isaacson); anena Ṅ

122 °tiṣṭhitātmā] *em.*; °tiṣṭhitātnā Ṅ

123 °traya°] *em.*; °trayaṃ Ṅ
